# A Library Screening Strategy Combining the Concepts of MS Binding Assays and Affinity Selection Mass Spectrometry

**DOI:** 10.3389/fchem.2019.00665

**Published:** 2019-10-04

**Authors:** Jürgen Gabriel, Georg Höfner, Klaus T. Wanner

**Affiliations:** Department of Pharmacy, Faculty of Chemistry and Pharmacy, Ludwig Maximilian University München, Munich, Germany

**Keywords:** MS Binding Assays, affinity selection mass spectrometry, library screening, hit identification, LC-MS

## Abstract

The primary objective of early drug development is to identify hits and leads for a target of interest. To achieve this aim, rapid, and reliable screening techniques for a huge number of compounds are needed. Mass spectrometry based binding assays (MS Binding Assays) represent a well-established technique for library screening based on competitive binding experiments revealing active sublibraries due to reduced binding of a reporter ligand and following hit identification for active libraries by deconvolution in further competitive binding experiments. In the present study, we combined the concepts of MS Binding Assays and affinity selection mass spectrometry (ASMS) to improve the efficiency of the hit identification step. In that case, only a single competitive binding experiment is performed that is in the first step analyzed for reduced binding of the reporter ligand and—only if a sublibrary is active—additionally for specific binding of individual library components. Subsequently, affinities of identified hits as well as activities of reduced sublibraries (i.e., all sublibrary components without hit) are assessed in additional competitive binding experiments. We exemplified this screening concept for the identification of ligands addressing the most widespread GABA transporter subtype in the brain (GAT1) studying in the beginning a library composed of 128 and further on a library of 1,280 well-characterized GAT1 inhibitors, drug substances, and pharmacological tool compounds. Determination of sublibraries' activities was done by quantification of bound NO711 as reporter ligand and hit identification for the active ones achieved in a further LC-ESI-MS/MS run in the multiple reaction monitoring mode enabling detection of all sublibrary components followed by hit verification and investigation of reduced sublibraries in further competitive binding experiments. In this way, we could demonstrate that all GAT1 inhibitors reducing reporter ligand binding below 50% at a concentration of 1 μM are detected reliably without generation of false positive or false negative hits. As the described strategy is apart from its reliability also highly efficient, it can be assumed to become a valuable tool in early drug research, especially for membrane integrated drug targets that are often posing problems in established screening techniques.

## Introduction

**Figure 1:**
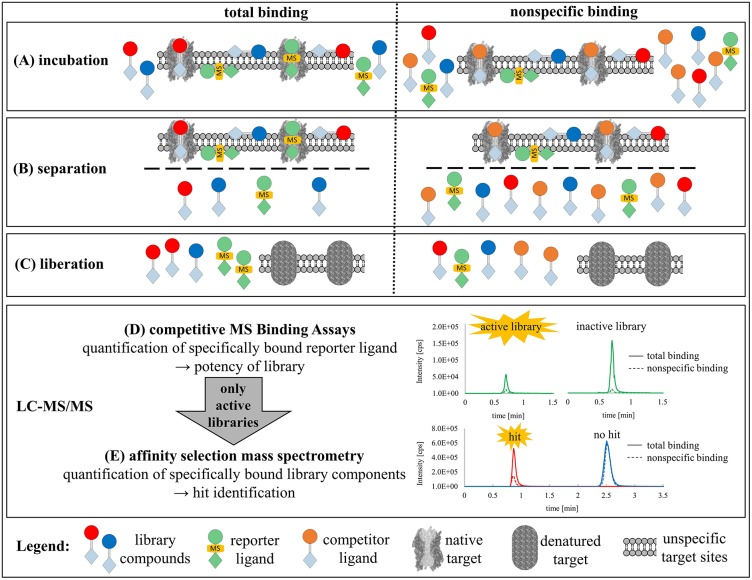
Combination of MS Binding Assays and affinity selection mass spectrometry (ASMS) for library screening. **(A)** Incubation of reporter ligand, target, sublibrary to determine the total binding of reporter ligand and library components as well as additionally an excess of competitor ligand for determination of non-specifically bound ligands. **(B)** Separation of bound from non-bound ligands by vacuum filtration. **(C)** Liberation of bound ligands with organic solvent and generation of samples for LC-MS analysis. **(D)** Competitive MS Binding Assay to determine library activity as a function of bound reporter ligand determined by LC-MS. **(E)** ASMS in the case of active sublibraries for the identification of the corresponding hits by means of LC-MS quantification of specifically bound library components as a function of total vs. non-specific binding.

The early-phase of drug discovery, where research is focused on exploration of hits and leads for a target of interest, is often a long, inefficient, and therefore expensive venture (Dimasi et al., 2003; Hughes et al., 2011). To keep these expenses as low as possible, efficient, and reliable techniques for the investigation of target-ligand interactions are essential. Despite the availability of a wealth of techniques for this purpose, great efforts are applied to improve data quality and to accelerate this time-consuming step in the drug discovery process. These techniques can be divided in two major categories. The first one is represented by functional assays showing an effect mediated by interaction of a ligand at a target binding site. Functional assays are, depending on the nature of the investigated target (such as e.g., enzymes, receptors, or ion channels), based on diverse read out principles such as cell proliferation, reporter gene expression, or downstream signaling effects as a consequence of ligand binding (Croston, 2002; Trivedi et al., 2010; Babbitt et al., 2015). Although these techniques can provide valuable information about the functional consequence triggered by a test compound at a target, they are also subjected to several limitations such as interferences with the recorded signal which may result in insufficient data quality or sometimes false positive results due to interactions at sites not associated with the addressed target—to mention just a few—and are furthermore, often associated with high efforts for their establishment or implementation. The second category, the so called ligand binding assays directly recording target-ligand binding, provide information about the affinity of a test compound for a target binding site. In comparison with functional assays, binding assays are less prone to interferences and are easier to establish, but have some drawbacks as well. They only reveal affinity but no functional activity and the throughput is often not as high as in functional assays. Ligand binding assays are very popular and particularly helpful in medicinal chemistry projects as they are best suited for the establishment of structure-activity relationships. So far, a vast number of approaches to monitor target-ligand binding with different detection techniques has been established (Fang, 2012). It is distinctly beyond the scope of this introduction to review them, therefore, only some of the most relevant ones should be mentioned here. In terms of affinity assessment, radioligand binding assays still represent the gold standard, due to their high sensitivity, excellent robustness, and simple implementation (Maguire et al., 2012). Especially the first argument is still an important one, as it allows to apply the target at rather low concentrations and does not require high sophisticated target expression or enrichment strategies. Despite these qualities, employment of “hot,” i.e., radioisotope labeled ligands is associated with disadvantages such as increased synthetic effort, hazards to human health, restrictions set by authorities, or expensive waste management. In order to overcome these limitations, several alternatives to avoid radioisotope labeled compounds, such as fluorescence, luminescence, surface plasmon resonance, or mass spectrometry (MS) based binding assays were established in the recent year (Geoghegan and Kelly, 2005; Zhu and Cuozzo, 2009; Stahelin, 2013; Höfner and Wanner, 2015; Stoddart et al., 2016). Although all of these alternatives provide specific strengths (and weaknesses) that should not be discussed in detail here, MS may be considered as particularly attractive detection principle as neither ligand nor target has to be modified or labeled for investigation of target-ligand interactions (Geoghegan and Kelly, 2005; Schermann et al., 2005; Höfner and Wanner, 2015). Even in the field of MS based binding assays, a wealth of concepts has been reported covering direct measurement of target-ligand complexes (Hofstadler and Sannes-Lowery, 2006) as well as monitoring bound ligands after their liberation from the target (Annis et al., 2007a; Höfner and Wanner, 2015) or even recording of ligands remaining non-bound (Hofner and Wanner, 2003) in presence of the target. In the context of the present study, we want to focus on two strategies of MS based binding assays, namely MS Binding Assays (Höfner and Wanner, 2015; Massink et al., 2015) and affinity selection mass spectrometry (ASMS) (Van Breemen et al., 1997; Zehender et al., 2004; Annis et al., 2007a; Jonker et al., 2011). MS Binding Assays are closely related to radioligand binding assays and share a lot of common features such as setup of the binding experiment as well as concentrations of ligands and targets. They are based on the use of a non-labeled reporter ligand instead of a ligand labeled with a radioisotope, which can be quantified highly sensitive by means of MS. Accordingly, binding experiments following this strategy can be performed as simple as radioligand binding experiments by incubation of the target with the reporter ligand (together with test compounds if necessary). MS Binding Assays require—just as radioligand binding assays—for termination of the binding experiment separation of the formed target-reporter ligand-complexes from non-bound reporter ligand which is typically achieved by filtration. In MS Binding Assays, subsequently to this separation step, the formerly bound reporter ligand is quantified typically by LC-ESI-MS/MS after its liberation and elution from the target-reporter ligand-complexes remaining on the filter with an organic solvent, whereas in radioligand binding experiments the bound reporter ligand remaining on the filter employed for separation is quantified by liquid scintillation counting (LSC). This strategy has been successfully applied to several membrane integrated drug targets such as neurotransmitter transporters (Zepperitz et al., 2006; Grimm et al., 2015; Ackermann et al., 2019), G protein-coupled receptors (Massink et al., 2015; Neiens et al., 2015; Chen et al., 2017), or ligand gated ion channels (Sichler et al., 2018). Furthermore, as a consequence of the superior selectivity of mass spectrometric detection (in comparison with LSC), MS Binding Assays enable binding experiments for multiple targets with corresponding selective reporter ligands simultaneously in the same binding sample (Schuller et al., 2017; Neiens et al., 2018). As the concept of MS Binding Assays has been established as alternative to radioligand binding assays, it is—in the same way as the latter—primarily suited for affinity determination of single compounds, but it is basically not restricted to this application. With the steadily increasing number of new chemical entities due to utilization of combinatorial chemistry for library synthesis (Aubé et al., 2014), but also to novel purification and exploitation techniques for natural resources (Kingston, 2011), it is more and more common to screen pooled compound libraries instead of single compounds. Competitive MS Binding Assays have also been demonstrated to be well-suited for this task as shown for the screening of synthesized hydrazone and oxime libraries addressing the GABA transporter 1 (GAT1). In these studies, the libraries were divided in sublibraries of four or eight compounds, which were screened by recording reporter ligand binding remaining in the presence of each sublibrary. In the further course of this work, such individually studied sets will always be referred to as “sublibraries.” Subsequently, the most potent sublibraries were subjected to deconvolution experiments, i.e., testing each sublibrary component alone in a single run to identify the corresponding hits (Sindelar et al., 2013; Kern and Wanner, 2019). The strength of this approach is a rather simple setup and an almost complete exclusion of false positive or false negative results due to monitoring of reporter ligand binding instead of directly identifying bound library components. The work required for deconvolution is in the case of focused libraries, where high hit rates are common, acceptable, but can be in the case of big libraries considerable. The other concept of MS based binding assays to be discussed here is gathered under the name ASMS. ASMS approaches primarily differ in their employed technique for the separation of bound and non-bound ligands (e.g., vacuum filtration, ultrafiltration, or size exclusion chromatography). In contrast to MS Binding Assays, ASMS—as already implicated by this term—enables direct identification of target-bound ligands by mass spectrometric detection. A particularly powerful and in the meantime well-established ASMS application is the automated ligand identification system (ALIS) (Annis et al., 2007b) which employs size exclusion chromatography for on-line separation of bound in form of ligand-target complexes from non-bound ligands followed by dissociation of target bound ligands on a reversed phase column and finally, detection (and identification) of the liberated ligands by time of flight MS. ALIS has been successfully employed primarily for soluble proteins, but in some cases also for membrane-bound protein targets and allows—with its outstanding high throughput capacity—to screen thousands of compounds per hour (Whitehurst et al., 2006; Kutilek et al., 2016; Walker et al., 2017). Additionally, Annis et al. applied the ALIS setup for determination of affinity rank-orders and also for affinity determination of ligands (Annis et al., 2007c). Despite these exceptional capabilities, ASMS remains as well-subject to several critical limitations. ASMS approaches are for example often prone to false positive hits which require elaborate hit evaluation experiments. An even more limiting, but at the same time fundamental drawback of this strategy is, however, that ASMS relies on the ability to detect (i.e., to identify) the employed test compounds by MS. This would mean that test compounds providing insufficient signal intensity under the chosen mass spectrometric conditions will be lost, almost fully independent of their affinity to the target. As the chance to identify a binding ligand following the ASMS concept is inherently coupled with the amount of target employed in the binding experiment, this approach is only feasible when considerably high target concentrations can be employed in the binding experiment and therefore, restricted to targets which are easily accessible from native materials or for which powerful expression and purification are available. Having said this, it was the aim of this study to develop an MS based library screening strategy which combines the strengths of competitive MS Binding Assays with those of ASMS, while at the same time overcoming the weaknesses of both concepts. To achieve this goal sublibraries' potencies should be assessed as a function of bound reporter ligand following the concept of competitive MS Binding Assays and in the case of an active sublibrary, the corresponding hit should be identified following the concept of ASMS without performing an additional binding experiment (Figure 1). In this way, it can be ascertained that no active ligands due to insufficient mass spectrometric sensitivity or low target concentration is missed due to the use of a reporter ligand in the first step (MS Binding Assays), while at the same time the elaborate deconvolution procedure can be avoided by following the ASMS concept. Thus, the entire library screening strategy presented in this study starts with a binding experiment by incubation of a first sample that contains the target—reporter ligand as well as library components—and defines total binding of reporter ligand and library components, respectively (Figure 1 “incubation,” left part). A second sample contains the same constituents (in the same concentrations) as the first one, but in addition a further ligand in excess to block the addressed target binding site serves as negative control. In this way non-specific binding of reporter ligand and library components, respectively, can be defined (Figure 1 “incubation,” right part). Then for both samples, bound and non-bound ligands (including the reporter ligand) are separated via vacuum filtration (Figure 1 “separation”) and subsequently, the formerly bound ligands (including the reporter ligand) are liberated from the target (Figure 1 “liberation”) and analyzed by LC-ESI-MS. In this way, initially in a first set of LC-MS runs, the sublibraries' activities can be easily assessed by quantification of the reporter ligand, as inhibition of its binding must be due to the presence of active components in sublibraries (Figure 1 “competitive MS Binding Assay”). For those sublibraries recognized as active, the corresponding hits can then be identified later on directly by mass spectrometric quantification of library components in an additional set of LC-MS runs, without performing another binding experiment (Figure 1 “affinity selection mass spectrometry”). By comparison of total and non-specific binding for all components of an active sublibrary, exclusively those components are identified as hits exhibiting specific binding, whereas false positive results can be omitted in this way. Finally, the activities of “reduced sublibraries,” i.e., sublibraries containing all library components except identified hit compounds, are again characterized in a competitive binding experiment. Thereby, false negative results caused by competitive effects due to the presence of more than one hit in a sublibrary or due to insufficient sensitivity of mass spectrometric quantification of bound library components can be ruled out. To prove the feasibility of this library screening concept, we chose the most widespread GABA transporter subtype in the brain GAT1, for which a competitive MS Binding Assay is already well-established. GAT1 is a membrane transport protein belonging to the solute carrier family SLC6, primarily located in the synaptic region on neurons, where it is primarily responsible for the termination of GABAergic signals (Scimemi, 2014). As several neurological disorders such as neuropathic pain (Gwak and Hulsebosch, 2011), sleep disorders (Gottesmann, 2002), schizophrenia (Lisman et al., 2008), epilepsy (Treiman, 2001), anxiety, or depression (Lydiard, 2003; Kalueff and Nutt, 2007) are associated with a GABAergic dysfunction, GAT1 represents an interesting drug target for several therapeutic indications. Therefore, efficient screening techniques are of great value by facilitating the development of new GAT1 inhibitors.

## Materials and Methods

### Chemicals and Reagents

LC-MS grade acetonitrile and methanol as well as tris(hydroxymethyl)-aminomethan (Tris) were purchased from VWR (Darmstadt, Deutschland). Ammonium formate was obtained from Fluka (Sigma Aldrich, Taufkirchen, Germany) in LC-MS ultra-grade purity. Water was purified by lab water purification system (Sartorius, Göttingen, Germany). NO711 and [^2^H_10_]NO711 were synthesized in-house and dissolved in DMSO (ACS Reagent Grade, Fisher Scientific UK, Loughborough, UK) to obtain 10 mM stock solutions. The 128 compound library (see below) consisted of well-known GAT1 inhibitors, synthesized in house (DDPM compounds, pre dissolved in 10 mM DMSO stock solution) as well as commercially available drug substances or organic chemicals, received from commercial providers. Chemical structures of DDPM compounds are shown in Table S1. All library compounds were dissolved in DMSO to yield 10 mM stock solutions. Compilation of the library: sublibrary A [(4-(4-chloro-phenyl)-piperidine-4-ol, 7-(dipropylamino)-5,6,7,8-tetrahydronaphthalen-1-ol hydrobromide (8-OH DPAT), DDPM2330, DDPM2565, chlorpromazine hydrochloride, doxepin hydrochloride, fenoterol hydrobromide, ketoprofen, meclozine dihydrochloride, metoclopramide hydrochloride, oxazepam, piroxicam, procaine hydrochloride, roxithromycin, sulpiride, telmisartan], sublibrary B [bifonazole, ciprofloxacin hydrochloride, DDPM2188, DDPM3138, glibenclamide, hydromorphone hydrochloride, lisinopril dihydrate, molsidomine, noscapine hydrochloride, pilocarpine nitrate, procainamide hydrochloride, prophyphenazone, reserpine, sulfaguanidine, sulfamethoxazole, triphenylamine], sublibrary C [aciclovir, amitriptyline hydrochloride, atropine sulfate, brucine, cetirizine hydrochloride, clomipramine, DDPM2029, DDPM2077, ethacridine lactate, indometacin, mepivacaine hydrochloride, (2-morpholin-4-ylmethyl-benzoimidazol-1-yl)-acetic acid, papaverine hydrochloride, promethazine hydrochloride, salbutamol sulfate, sertraline hydrochloride], sublibrary D [ambroxol hydrochloride, antazoline hydrochloride, biperidene hydrochloride, clotrimazole, DDPM2187, DDPM2473, diphenhydramine hydrochloride, diltiazem hydrochloride, lidocaine, meloxicam, ofloxacin, physostigmine salicylate, propranolol hydrochloride, ranitidine hydrochloride, sulfisomidine, tianeptine], sublibrary E [(4-(4-(dimethylamino)styryl)-N-methylpyridinium iodide (ASP^+^), atenolol, benzylpenicillin potassium, chlordiazepoxide hydrochloride, DDPM1349, DDPM1981, drofenine hydrochloride, isoprenaline sulfate dihydrate, meprobamate, morphine hydrochloride, nalidixic acid, phenylbutazone, terfenadine, tetracaine hydrochloride, tolbutamide, verapamil hydrochloride], sublibrary F [(2-(4-methyl-1,4-diazepan-1-yl)benzoic acid, baclofen, chloroquine phosphate, DDPM2009, diazepam, ditolylguanidine, haloperidol, ipratropium bromide, metoprolol tartrate, moxifloxacin hydrochloride, naphazoline hydrochloride, phenobarbital, pimozide, scopolamine hydrobromide, tetracycline hydrochloride, tramadol hydrochloride], sublibrary G [buspirione hydrochloride, clonidine hydrochloride, cimetidine, DDPM3139, imipramine hydrochloride, metipranolol, 8-{3-[bis(4-fluorophenyl)amino]propyl}-3-methyl-1-phenyl-1,3,8-triazaspiro[4.5]decan-4-one maleate (PH014034), quinine hydrochloride, piretanide, ramipril, riboflavin, strychnine nitrate, tiabendazole, trifluoperazine, trimethoprim, xylometazoline hydrochloride], sublibrary H [(3-(2-methyl-1H-imidazol-1-yl)benzoic acid, acetazolamide, captopril, chloramphenicol, chlortalidone, diclofenac sodium, etacrynic acid, furosemide, hydrochlorothiazide, mefenamic acid, methyl orange, naproxen, lauryl maltoside, niclosamide, nitrazepam, phenytoin]. Tocris ScreenPlus library (1280 compounds, 10 mM in DMSO) was purchased from Bio-Techne (Wiesbaden-Nordenstadt, Germany).

### LC-ESI-MS/MS Instrumentation

LC-ESI-MS/MS was performed using a QTRAP5500 triple quadrupole mass spectrometer with a TurboV-ion source (Sciex, Darmstadt, Germany) coupled to an Agilent 1260 HPLC system (G 1322A Degasser, binary pump G1312B, oven G1316A, Agilent, Waldbronn, Germany) and a SIL-20A/HT autosampler (Shimadzu, Duisburg, Germany). Autosampler settings were set as follows: rinsing volume 200 μL (acetonitrile/water 50:50, v/v), needle stroke 52 mm, rinsing speed 35 μL/s, sampling speed 5.0 μl/s, purge time 1.0 min rinse dip time 0 s and rinse mode was set to “before and after aspiration.” Data acquisition and analysis was carried out with Analyst 1.6.3 software.

### Chromatography

For LC, a Purospher Star RP18e column (55 × 2 mm, 3 μm, Merck KGaA, Darmstadt, Germany) protected with a C-18 Guard Cartridge column (4 × 2 mm, Phenomenex, Torrance, CA, USA) and two in-line filters (0.5 μm and 0.2 μm, IDEX, Oak Harbor, WA, USA) was used as stationary phase. Column oven temperature was maintained at 25°C. Unless stated otherwise, the samples to be analyzed were dissolved in ammonium formate buffer (10 mM, pH 7.0) and methanol in a ratio of 30:70 (v/v). For recording of the reporter ligand NO711 by LC-MS, an isocratic elution mode with 10 mM ammonium formate buffer at pH 7.0 and acetonitrile at a ratio of 50:50 (v/v) was employed. The injection volume was set to 10 μL at a flow rate of 350 μL/min. For hit identification by LC-MS, a gradient elution at a flow rate of 450 μL/min and 25°C column temperature was performed according to the following conditions: after starting with 10 mM ammonium formate buffer (pH 7.0)/ acetonitrile at a ratio of 60/40 (v/v) the mobile phase was changed to a ratio of 20/80 (v/v) from 0.01 to 0.05 min and held until 3.5 min. Between 3.50 and 3.55 min the ratio was changed back to the starting conditions and held until 9 min. The injection volume was set to 40 μL.

### Compound-Dependent MS Parameters

For quantification of reporter ligand binding, the mass transitions *m/z* 381 → 180 and *m/z*391 → 190 for NO711 and [^2^H_10_]NO711, respectively, were used as described recently (Zepperitz et al., 2006), applying the following compound-dependent MS parameters: declustering potential 80 V, entrance potential 10 V, collision energy 25 V, and collision cell exit potential 18 V. For hit identification, the compound-dependent MS parameters of precursor ion and the eight most intensive product ions of each library compound were optimized via “Quantitative Optimization.” For this purpose, for each sublibrary a solution containing all components in a concentration of 20 nM was prepared in 10 mM ammonium formate buffer at pH 7.0/acetonitrile at a ratio of 20:80 (v/v). These solutions were directly infused into the ESI-source at a flow rate of 7 μL/min via the integrated syringe pump. Detailed instrument settings (see Table S2) as well as the compound-dependent MS parameters for every compound obtained thereby are presented in the Tables S3, S4. Compounds of sublibrary H were investigated in the negative, all others in the positive ionization mode.

### Source-Dependent MS Parameters

For quantification of the reporter ligand, the following source settings were applied: temperature 650°C, ion-spray voltage 3,000 V, curtain gas (N_2_) 30 psi, auxiliary gas (N_2_) 40 psi, nebulizing gas (N_2_) 50 psi, and collision gas (N_2_) “high,” dwell time 100 ms, mass resolution unit. For hit identification under positive ionization conditions, the settings were as following: source temperature 650°C, ion-spray voltage 2,000 V, curtain gas (N_2_) 28 psi, auxiliary gas (N_2_) 40 psi, nebulizing gas (N_2_) 50 psi and collision gas (N_2_) “high,” dwell time 20 ms (in case of libraries consisting of 64 compounds 5 ms), mass resolution unit. For hit identification under negative ionization conditions the settings were as following: source temperature 650°C, ion-spray voltage −2,500 V, curtain gas (N_2_) 30 psi, auxiliary gas (N_2_) 50 psi, nebulizing gas (N_2_) 60 psi and collision gas (N_2_) “mid.” Dwell time 20 ms, mass resolution unit.

### Evaluation of the Library Components' Mass Transitions by LC-ESI-MS/MS

For each sublibrary, a matrix blank (see generation of matrix) as well as solutions containing all sublibrary components in concentrations of 1 and 10 nM, respectively, in matrix were analyzed with the gradient LC-ESI-MS/MS method employing all the mass transitions with their optimized potentials (obtained as described above) for the corresponding precursor and product ions. Based on the comparison of the MRM chromatograms obtained thereby, the most appropriate mass transitions for quantification of the corresponding compounds were selected (according to the highest signal to noise ratio for 1 nM matrix standards).

### Membrane Preparation

Membranes of HEK293 cells stably expressing mGAT1 were prepared as described previously and stored at −80° C (Zepperitz et al., 2006). Per 96 well-plate, an aliquot of 2 mL was thawed and diluted in 20 mL 0.9% (w/v) sodium chloride solution. After 20 min centrifugation at 20,000 rpm/4°C (Sorvall, rotor SS34, Thermo Fisher, Waltham, US), the pellet was resuspended (Polytron, PT2000, Kinematica AG, Littau, Switzerland) in incubation buffer (50 mM Tris,1 M NaCl, adjusted with citric acid to pH 7.1) resulting in a protein content of ~100 μg/mL, determined according to Bradford.

### MS Binding Assay

All binding experiments were performed in a polypropylene 96-well plate (1.2 mL well volume, Sarstedt, Nümbrecht, Germany) in analogy to the procedure recently described (Zepperitz et al., 2006). In all cases triplicate samples were prepared in incubation buffer (see above) containing the GAT1 membrane preparation (protein content 20–30 μg/well, yielding a final GAT1 concentration of about 3 nM) and NO711 (final concentration 10 nM) in a total volume of 250 μL. One set of samples additionally contained the sublibraries (each component in a final concentration of 1 μM) for determination of total reporter ligand binding in presence of the sublibraries and total binding of the sublibrary components. Analogously, a second set of samples additionally contained the sublibraries (each component in a final concentration of 1 μM) together with 100 mM GABA for determination of non-specific reporter ligand binding and non-specific binding of the sublibraries' components. Furthermore, a single triplicate without any additions served as a control for total reporter ligand binding in the absence of any other ligand. All samples were incubated for 40 min at 37°C using a plate shaker incubator (Stuart Microtitre SI505, Bibby Scientific Limited, Staffordshire, Great Britain). The incubation was stopped by vacuum filtration using a multi well-plate vacuum manifold (Pall, Dreieich, Germany) in combination with a 96-well glass fiber filter plate (AcroPrep Advance, glass fiber, 1.0 mm, Pall, Dreieich, Germany) pretreated with 200 μL 0.9% (w/v) sodium chloride solution. Aliquots of 200 μL per well were transferred to the filter plate with a 12-channel pipet and subjected to vacuum filtration. Subsequently, the filter plate was washed four times with 150 μL ice-cold 0.9% (w/v) sodium chloride solution and dried at 50°C for 60 min. Afterwards, the filters with the remaining target and target-bound ligands were exposed to 100 μL methanol containing [^2^H_10_]NO711 (1.4 nM) as internal standard per well for 15–20 s and then eluted into a deepwell plate by application of vacuum. This step was repeated twice resulting in a total elution volume of 300 μL. Finally, a volume of 130 μL ammonium formate buffer (10 mM, pH 7.0) was added per well. In the end, the resulting samples were equally split into two 96 well-plates and sealed with aluminum foil. The first 96 well-plate was immediately subjected to quantification of reporter ligand by LC-ESI-MS/MS. The second 96 well-plate was stored at −20°C and only examined for target-bound library components by LC-ESI-MS/MS recording the corresponding mass transitions (see Evaluation of the library component's mass transitions by LC-ESI-MS/MS) in the case of active sublibraries.

### Generation of Matrix

Samples of 250 μL in incubation buffer (50 mM Tris, 1 M NaCl, pH 7.1) containing GAT1 membrane preparation (protein content 20–30 μg/well) per well without any ligand were exactly processed according to the procedure of “MS Binding Assay” described above up to the elution step. Elution was done with pure methanol (without internal standard) and the resulting methanolic eluate was stored at −20°C. To prepare the required matrix standards, 130 μL ammonium format buffer (10 mM, pH 7.0), containing internal standard and library components, or in case of blank matrix sample, without both, were added to aliquots of 300 μL methanolic eluate.

### Data Analysis

Based on the calibration function (established for each binding experiment), the concentrations of NO711 resulting from the corresponding binding samples were calculated using Analyst v.1.6.3 (Sciex, Darmstadt, Germany) as described previously (Zepperitz et al., 2006). Remaining bound reporter ligand was calculated as the percentage of the specifically bound reporter ligand in presence of a sublibrary compared to specifically bound reporter ligand in the absence of a sublibrary. All binding experiments were performed in triplicates and results are given as means ± SD. For hit identification, all chromatographic parameters and normalized peak areas (area sublibrary component/area internal standard) of each individual sublibrary component in the corresponding MRM chromatograms of samples representing total and non-specific binding were calculated via Analyst v.1.6.3 (Sciex, Darmstadt, Germany). Consequently, all library components showing significant specific binding as difference of the normalized areas of total and non-specific binding (one site student *t*-test, *n* = 3, CL = 97.5%) were classified as hits.

## Results and Discussion

### Basic Considerations Before Implementation of the Library Screening Concept

For a methodical combination of the approaches of competitive MS Binding Assays and ASMS for the screening of ligands addressing GAT1, it is necessary to consider some basic points regarding setup and conditions in the GAT1 binding experiments, LC-MS analytics as well as potencies of the ligands to be identified. First of all, the already established filtration based competitive GAT1 MS Binding Assays, which were already utilized for the screening of hydrazone and oxime compound libraries in our group, should serve as a basis for the development of the new assay (Zepperitz et al., 2006).

If vacuum filtration as separation technique is intended to be employed for the separation of bound ligands from non-bound ligands, it has to be ensured that only minor amounts of the target bound ligand are lost due to dissociation of the target-ligand complex during the necessary washing step.

In the textbooks, it is often stated that this requirement is fulfilled when the affinity of the ligand toward the target expressed as *K*_d_-value equals 10 nM or less (Bennett and Yamamura, 1985). However, target-ligand complex dissociation is more a question of the *k*_*off*_–rate than of *K*_d_. This means that ligands with higher *K*_d_-values can also be employed in filtration based binding assays as long as the corresponding *k*_*off*_–rate is low enough. NO711, the reporter ligand used in our GAT1 MS Binding Assays, is an example for a compound showing only moderate affinity, characterized by a *K*_d_-value of 23.6 nM toward GAT1, but with a comparably low *k*_*off*_–rate of about 1.5 × 10^−3^ s^−1^ (Zepperitz et al., 2006). Accordingly, it proved to be excellently suited as reporter ligand for GAT1 in filtration based MS Binding Assays for which purpose it has been extensively applied for more than one decade. Under particularly favorable conditions, such as a low *k*_*off*_–rate together with a very short time period for the washing process at low temperature, reporter ligands with *K*_d_-values even beyond 100 nM have been used in filtration based binding assays, as for example described for [^3^H]TCP (Katz et al., 1997), [^3^H]PCP (Eldefrawi et al., 1982), or [^3^H]imipramine (Arias et al., 2010) addressing the nACh receptor. Library components binding to the target during a binding experiment, which are to be directly identified by ASMS after filtration, are subject to dissociation during washing in the same way as described above for the reporter ligands. However, in these cases losses of bound ligands can be tolerated to a higher extent, as far as a sufficient amount of the formerly target-bound ligand is left for detection by LC-MS. Therefore, we considered affinities in the high nM range close to 1 μM as the ultimate limit compounds should have to be detectable under the conditions of our filtration based GAT1 MS Binding Assays. Taking into account the situation of a competitive binding experiment applying the reporter ligand NO711 in a concentration of 10 nM and the library components all in a concentration of 1 μM, inhibition of reporter ligand binding due to a single library component down to a level of 50% can be attributed to an affinity (i.e., a *K*_i_-value) of about 700 nM for this compound (according to the equation of Cheng-Prusoff, for details see Table S5). Following these considerations, we set the concentration for the individual test compounds in the libraries to 1 μM.

Another fundamental parameter in binding assays is the concentration of the target, which is typically kept below 0.1 *K*_d_ of the reporter ligand in radioligand as well as in MS Binding Assays to avoid depletion of the reporter ligand (Hulme and Birdsall, 1992). This condition is, however, in clear contrast to the need for target concentrations in affinity selection approaches that should be as high as possible to facilitate hit identification. According to our experiences with quantification of various reporter ligands in MS Binding Assays by means of LC-MS/MS employing triple quadrupole mass spectrometers, we considered it feasible to quantify a vast majority of possible library components down to a concentration level of at least 1 nM in the matrix generated in the binding experiment. Therefore, we expected that a GAT1 concentration of about 0.1 *K*_d_ should be high enough to achieve our aims and, hence, decided to employ GAT1 at a concentration of about 3 nM (i.e., 0.125 *K*_d_). Accordingly, for a library component reducing NO711 binding to 50% at a concentration of 1 μM, the equilibrium concentration of the corresponding GAT1-ligand complex formed by this compound can be estimated to be about 1.5 nM (for details see Table S5) in the binding experiment. It has, however, to be taken into account, that the actual concentration of such a library component in the final sample to be subjected to LC-MS will be distinctly lower than 1.5 nM due to the above mentioned issue of dissociation during the washing step following filtration. It is obvious that library components with higher affinities will lead to higher equilibrium concentrations of target-ligand complexes (maximally approaching the GAT1 concentration of about 3 nM) and that such compounds are in tendency less prone to losses due to dissociation. In contrast to the case with only one active component in the library, the situation changes, when more active library components are present in the binding experiment. For two ligands for example, both with an affinity (*K*_i_) of about 700 nM (i.e., an IC_50_ of 1 μM), the equilibrium concentrations for the corresponding target-ligand complexes can be estimated to about 1 nM for both ligands, and it is obvious that target-ligand concentrations will decrease further when even more active components are present. The situation gets particularly unfavorable, when a weak binder and a strong binder are present in same library, for example one with an affinity of about 700 nM (i.e., an IC_50_ of 1 μM) and one with a 100 times higher affinity. In this case, the strong binder will suppress binding of the weak binder, resulting in an equilibrium concentration of the corresponding target-ligand complex of about 30 pM (see Table S5). As this concentration will further decrease during washing due to dissociation, the risk to not identify this compound in the affinity selection step is high. According to these considerations and the inherent issue that there might be compounds in the library hardly to quantify by MS, it is clear that under the envisaged screening conditions including detection by MS, false negative results can hardly be avoided in the affinity selection based hit identification step. In combination with the competitive MS Binding Assay, however, such cases can be easily identified, as inhibition of reporter ligand binding unequivocally indicates the presence of hits (as analogously, no inhibition of reporter ligand binding indicates the absence of hits). In the context of a weak binder (with an IC_50_ of 1 μM) that might have been missed in the affinity selection step, due to the presence of strong binder—as mentioned above—an additional subsequently performed competitive binding experiment with the corresponding sublibrary from which the strong binder identified before as hit (here referred to as “reduced sublibrary”) has been removed, will indicate the presence of this binder (reduction of ligand binding below 50%). Therefore, the weak binder initially missed in the first affinity selection step would be detected in the subsequent one.

HPLC coupled mass spectrometric detection is a further essential point in this concept. As we intended to use the already established GAT1 MS Binding Assays with a triple quadrupole mass spectrometer based quantification of the reporter ligand as starting point for this screening approach, it was obvious to employ this type of mass spectrometer also for the affinity selection step. Making use of this instrument type, we tried to benefit from its unsurpassed sensitivity for quantification of known compounds by running it in the multiple reaction monitoring (MRM) mode. To fully exploit the potential of the MRM mode, it is necessary to tune the mass spectrometer in a way that the corresponding mass transitions of all library components can be detected with maximum sensitivity. Although modern triple quadrupole mass spectrometers can provide analyte specific mass transitions together with a set of optimized potentials in automated procedures, the efforts for this step are considerable, when high numbers of compounds have to be investigated. In the presented concept, this additional effort is, however, only necessary, when inhibition of reporter ligand binding indicates the presence of a hit in a sublibrary. Quantification of target-bound reporter ligand and target-bound library components can basically easily be performed in a single LC-MS/MS run. Nevertheless, we decided to separate both steps, as that way the above mentioned efforts to establish MRM based quantification the library components can be minimized. This means that a first LC-MS/MS run is performed for quantification of the reporter ligand NO711 (under already established conditions). In the case of an active sublibrary, the corresponding binding samples are subjected to quantification of all individual library components in a second LC-MS/MS run, the conditions of which have then to be established. In the context of mass spectrometric detection, it should be finally mentioned, that it is surely also possible to use other instrument types such as ion traps, orbitraps, or TOFs, especially for the affinity selection step, but possibly also for quantification of the reporter ligand.

Important points to be considered when planning a screening method are the size of the sublibraries that can be used and the hit rates that are expected. The sizes of sublibraries employed in screening campaigns are often very different, depending on the aim of a screening project. As a size of thousand members and even more is quite common, the issue, how many compounds an individual set should contain is important. Certainly it is in principle possible to quantify at least hundreds of compounds simultaneously in a single LC-MS/MS with triple quadrupole mass spectrometers, sublibraries consisting of so many members are not really convenient to handle, when conditions for an MRM based quantification of the library components have to be established. With respect to the binding experiment, the size of a sublibrary appears—at a first glance—hardly restricted, and obviously expenditure of material (e.g., target, consumables etc.) and time can be saved when big sublibraries are employed. It has to be taken into account, however, that a high number of components will enhance the probability, that weak inhibition of individual binders will sum up to a significant overall inhibition of reporter ligand binding. This means that an inhibition of reporter ligand binding below 50% (i.e., the activity criterion chosen for the assessment of sublibraries) can also be due to numerous weak binders, which would cause severe efforts for their quantification in the affinity selection step. Actually, also the hit rate of library screening will distinctly influence the efficiency of the screening process related with size of the sublibrary, as each “active” sublibrary has to be further investigated in the affinity selection step, according to our concept. As the “activity” criterion applied to the results determining when a library is active or not can be adjusted at will, the described screening strategy provides a high degree of flexibility. Considering the above mentioned arguments, we chose a sublibrary size of 16 components as a compromise, but in order to demonstrate that the concept is not restricted to such a small number only, we exemplarily investigated a sublibrary comprising 64 components.

### Investigation of a Deliberately Compiled Test Library Consisting of 128 Components

#### Library Compilation

For implementation of our novel library screening concept, we compiled a library with 128 small molecule compounds (with a molecular weight from 201 to 837 Da) including 116 well-known drug substances or organic chemicals as well as 11 known GAT1 inhibitors and one non-selective low-potent GAT inhibitor with the intention to cover a broad range of structural diversity which is reflected by log D_pH7.0_ values from about −3 to almost 8 (calculated by MarvinSketch software) of the selected compounds. With regard to our aim to identify ligands with an IC_50_-value of about 1 μM or better, we chose—out of the pool of GAT1 inhibitors synthesized in our group—compounds with affinities ranging from p*K*_i_-values of 5.94 to 8.13 (data from in-house MS Binding Assays) and differing as far as possible in their structure. Almost all highly potent GAT1 inhibitors known so far share three common structural motifs, namely a heterocyclic amino acid and a lipophilic aryl-moiety both connected via a linker consisting of a hydrocarbon chain with different length, which may contain heteroatoms and multiple bonds. Accordingly, we tried to include test compounds with variations of all three structural motifs in the set of selected GAT1 inhibitors (Table S1). This set of 128 compounds was divided in eight sublibraries each consisting of 16 components whereby the following considerations were taken into account. First, the sublibraries were composed in a way that different activity patterns arose. Thus, the sublibraries A-E contained two, sublibrary F one, and sublibraries G and H no GAT1 inhibitor (see experimental section “Chemicals and Reagents”). Secondly, we compiled the sublibraries in a way that A-G could be investigated by using electrospray ionization in the positive and H in the negative ion mode in the MS quantification step, respectively. Thirdly, for the sake of simplicity, we grouped the compounds in a way that mass-encoded sublibraries resulted. That way the selection of appropriate mass transitions for the individual library components and accordingly, optimized parameters was facilitated.

#### Determination of Mass Transitions for the Library Components

As discussed in the basic consideration chapter above, we intended to use the MRM mode of a triple quadrupole mass spectrometer for quantification of total as well as non-specific binding of each component of sublibraries identified as active. Therefore, the compounds' mass transitions as well as the corresponding compound-dependent MS parameters were required for the MS analysis. Basically, only active sublibraries would have to be further investigated according to our described screening concept. In order to estimate the efforts and the efficiency for determination of mass transitions for the individual sublibrary components suited for further LC-MS/MS analysis, however, we decided to study all eight sublibraries of our deliberately compiled library at this step. Furthermore, we wanted to ensure that as much as possible of the selected compounds of the whole library can be detected in the MRM mode and therefore reflecting a broad spectrum of chemical structures during the following development of an “universal” chromatography, finally enabling quantification of the library components by LC-ESI-MS/MS. The desired mass transitions together with the corresponding compound-dependent MS parameters of the test compounds were obtained via the automated tuning mode of the mass spectrometer during infusion of solutions comprising each component of a sublibrary (i.e., 16 components) in a concentration of 20 nM. As preliminary experiments showed that automatic optimization of the intensity of the mass transitions of the eight most intensive fragment ions for each parent ion proved to be a good compromise between the amount of work and the quality of the obtained mass transitions for each library component (see below), all mass transitions (as described in Table S3) were determined accordingly.

#### Quantification of Library Components by LC-ESI-MS/MS

Based on the mass transitions and compound-dependent MS parameters obtained for the test compounds, an LC-ESI-MS/MS method suited for quantification of each library component should be established. We found the following gradient conditions using the same C18 stationary phase as for the quantification of reporter ligand, appropriate for our purpose: For the start of the chromatography, a solvent mixture with a ratio of 60/40 (v/v, 10 mM ammonium formate, pH 7.0/acetonitrile) had to be used, which after 0.01 min had to be rapidly changed to a solvent ratio of 20/80 thus reducing the aqueous component. This solvent ratio remained unchanged until the run time amounted to 3.5 min. Then, the solvent ratio was rapidly switched back to original conditions (60/40 v/v). This solvent ratio was applied for 5.5 min thus leading to a total runtime of 9.0 min for each sample injection (for more details see Supplementary Material).

**Figure 2:**
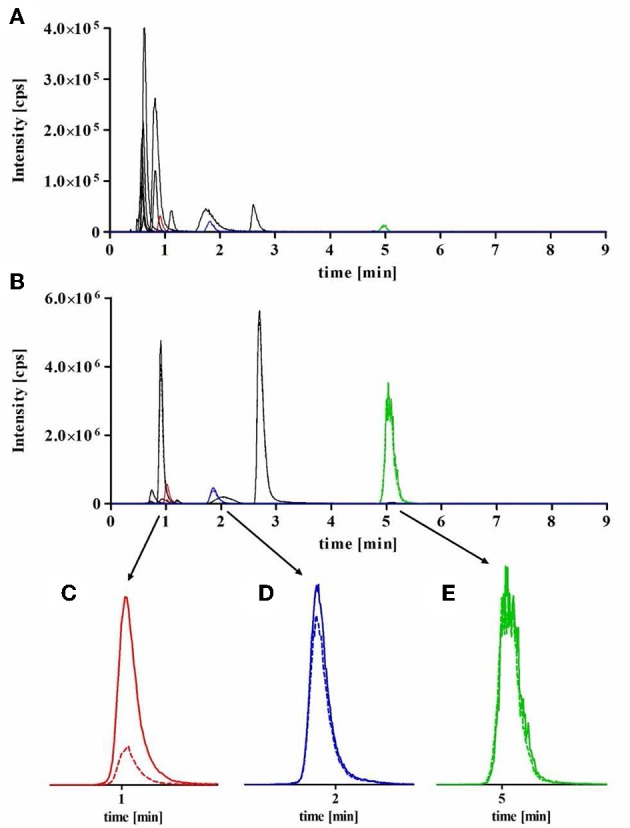
Representative MRM chromatograms obtained for sublibrary A during hit identification. For HPLC a Purospher Star RP18e column (55 × 2 mm, 3 μm) in combination with 10 mM ammonium formate buffer (pH 7.0) and acetonitrile (gradient conditions see “materials and methods,” injection volume 40 μL) at a flow rate of 450 μL/min and a column temperature of 25°C was used. **(A)** matrix standard containing each library component sample at a concentration of 1 nM, **(B)** total (full line) and non-specific binding (dotted line) samples (same binding samples as used in Figures 3C,D) at a concentration of 1 μM for each library component. Nonspecific binding was measured in presence of 100 mM GABA. Enlarged MRM chromatograms of **(C)** GAT1 inhibitors DDPM2565 (*m/z* 403 → 154) and **(D)** DDPM2330 (*m/z* 418 → 191), both showing significant specific binding toward GAT1 and **(E)** meclozine (*m/z* 391 → 201) as a representative compound without specific binding toward GAT1, note that full and dotted lines are in this case so close together that they can hardly be distinguished.

After optimization of the source-dependent MS parameters for these chromatographic conditions, MRM chromatograms for all sublibraries were recorded based on the mass transitions determined before (see above). For an unambiguous assignment of the peaks in the MRM chromatograms of the sublibraries (showing eight mass transitions per compound) to the corresponding library components, it proved to be helpful to compare for each sublibrary a set of three chromatograms, i.e., a matrix blank as well as two samples containing all the components of a sublibrary at a concentration of 1 and 10 nM, respectively, in the matrix of the binding experiment. Furthermore, we calculated the signal to noise ratios (S/N) for the most promising mass transitions of each compound and selected those providing the highest S/N ratios for final quantification. In this way, we could detect 122 of the 128 compounds based on the mass transitions determined before, presenting a success rate of 95%. A representative MRM chromatogram of sublibrary A is shown in Figure 2A and detailed results of all sublibraries are presented in Table S6. At this point, it is furthermore worth mentioning that all these compounds could be detected with an S/N ratio distinctly >5, which is the commonly accepted criterion for the lower limit of quantification (LLOQ). The compounds not detectable by MS (in the MRM mode) under these conditions were, triphenylamine, bifonazole, 4-(4-chloro-3-methyl-phenyl)-piperidin-4-ol, phenobarbitone, riboflavin, and clotrimazole. Surely, further efforts could be made for these compounds to find more appropriate mass transitions, but we deliberately decided to rely only on the automated tuning procedure to keep this step as simple as possible. Since [^2^H_10_]NO711 at a constant concentration of 1 nM is always present in the samples to be subjected to LC-ESI-MS/MS as internal standard for the quantification of bound reporter ligand NO711, we employed this compound also as internal standard, for quantification of our library components. In this way, the corresponding normalized areas could be calculated for all individual compounds based on their peak areas in relation to the peak area of the internal standard. As all compounds were investigated at the same concentration as the internal standard, i.e., at 1 nM, these normalized areas reflect at the same time the relative response factors (RRF) which are also summarized in Table S6. To sum up, it can be stated that the established method for LC-ESI-MS/MS quantification employing a triple quadrupole mass spectrometer is well-suited for quantification of a broad diversity of small molecule compounds down to a concentration of at least 1 nM either using the positive or the negative ionization mode.

#### Activity Assessment by Means of Competitive MS Binding Assays

**Figure 3:**
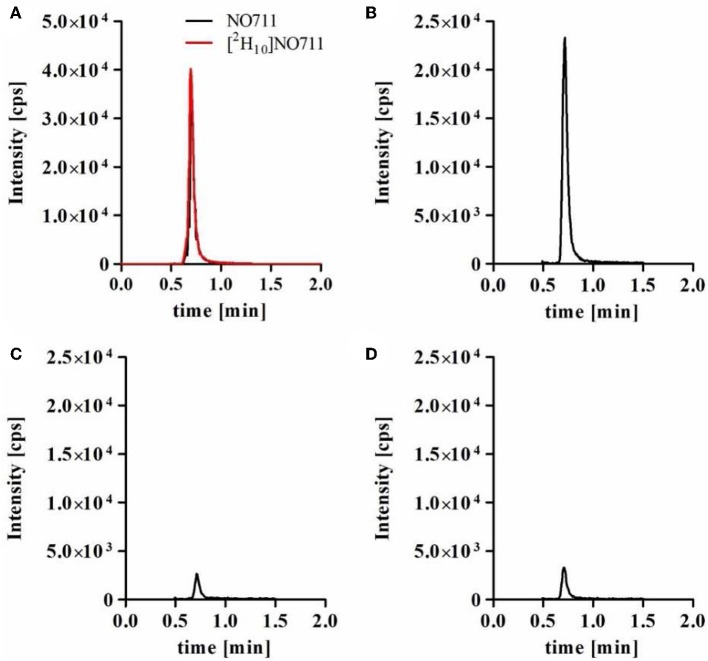
Representative MRM chromatograms obtained in competitive MS Binding Assay investigating sublibrary A. For HPLC, a Purospher Star RP18e column (55 × 2 mm, 3 μm) in combination with 10 mM ammonium formate buffer (pH 7.0) and acetonitrile (ratio 50:50, v/v) at a flow rate of 350 μL/min, a column temperature of 25°C and an injection volume of 10 μL. **(A)** calibration standard containing 1 nM NO711 (*m/z* 381 → 180) and 1 nM [^2^H_10_]NO711 (*m/z* 391 → 190), **(B)** total binding of NO711. **(C)** Non-specific binding of NO711 was determined in the presence of sublibrary A at a concentration of 1 μM and 100 mM GABA and **(D)** remaining binding of NO711 only in presence of sublibrary A at a concentration of 1 μM. All samples contained [^2^H_10_]NO711 (*m/z* 391 → 190) as internal standard at a concentration of 1 nM, but for simplification of the obtained chromatograms the corresponding trace (*m/z* 391 → 190) is only shown in **(A)**. Note that these chromatograms **(C,D)** are based on the same samples as the chromatograms depicted in Figure 2B.

**Figure 4:**
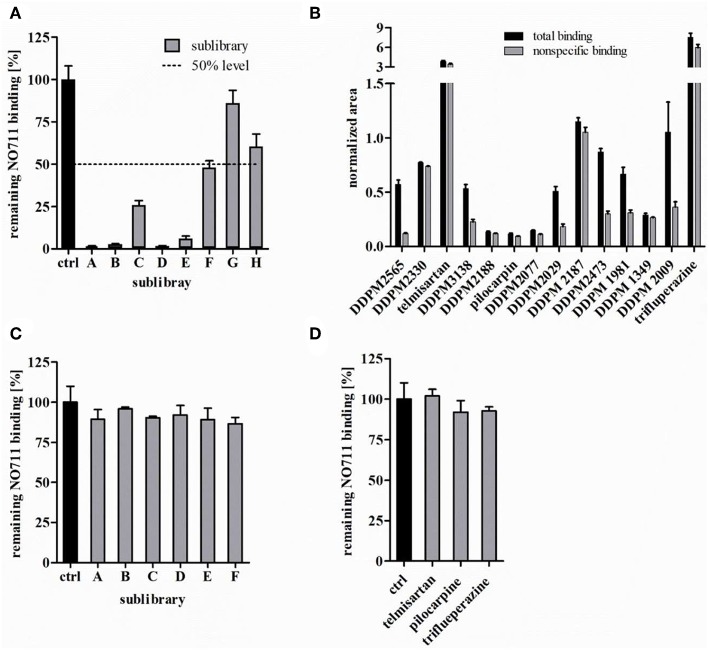
Screening of the deliberately compiled 128 compound library. **(A)** Remaining specific NO711 binding in % in presence of the sublibraries A-H (concentration 1 μM each compound) using isocratic LC-ESI-MS/MS. Dashed line indicates the defined limit for further investigation. **(B)** Total and non-specific binding of all identified hits at a concentration each of 1 μM for each sublibraries component. Non-specific binding was determined in the presence of 100 mM GABA. **(C)** Remaining NO711 binding in % in presence of the reduced sublibraries A-F (each sublibrary compound 1 μM) and **(D)** in presence of the putative false positive hits (each compound 1 μM). Reported values represent means ± SD (*n* = 3).

After establishment of an LC-ESI-MS/MS quantification method enabling highly sensitive quantification of the vast majority of library components, the described screening concept should be applied to screening of the 128 compound library for ligands addressing the NO711 binding site of GAT1. According to this concept, the activities of the eight sublibraries (A-H) had to be initially assessed. For this purpose, competitive GAT1 MS Binding Assays were performed completely in analogy to the procedure recently described (Zepperitz et al., 2006), always employing the reporter ligand NO711 at a concentration of 10 nM and mGAT1 at a concentration of about 3 nM. In the first set of samples of the MS binding experiments in addition to the reporter ligand, the sublibraries were contained with each component in a final concentration of 1 μM for determination of total reporter ligand binding in presence of the sublibraries and noteworthy, also for determination of total binding of the sublibrary components in the subsequent affinity selection step (only necessary in the case of an active sublibrary). Analogously, a second set of samples contained in addition to the sublibraries (with each component in a final concentration of 1 μM) 100 mM GABA for determination of non-specific reporter ligand binding and again, also for determination of non-specific binding of the sublibrary components (so far as necessary). These samples generated in the competitive MS binding experiment were analyzed for remaining reporter ligand binding by means of the isocratic LC-ESI-MS/MS method enabling quantification of NO711. A representative set of chromatograms obtained in this way, is exemplarily shown for sublibrary A in Figure 3. From these chromatograms, the percentages of specific NO711 binding remaining in presence of each sublibrary in relation to a control (only containing reporter ligand and target but not any other ligand = 100%) given in Figure 4A were determined. As expected, sublibraries A-E, all including two known GAT1 inhibitors, diminished remaining NO711 binding clearly under the 50% limit. Sublibrary F containing only 1 weak GAT1 inhibitor (p*K*_*i*_ of 5.94) was able to reduce reporter binding to 48%, whereas sublibraries G and H—not comprising a known GAT1 inhibitor—diminished NO711 binding only to 86 and 60%, respectively.

#### Hit Identification in Active Sublibraries by Means of ASMS

According to the screening concept, already described above in detail, hit identification by mass spectrometric quantification of bound sublibrary components has only to be accomplished for those sublibraries diminishing remaining reporter ligand binding below 50%. In order to investigate and to evaluate the envisaged hit identification procedure as systematically as possible, however, we had decided to evaluate all sublibraries. Accordingly, the samples, representing total and non-specific binding generated in the binding experiments before, were again analyzed by LC-MS (under the established gradient conditions), now recording MRM chromatograms for the individual library components to identify those library members showing a specific binding toward the NO711 binding site of GAT1 (i.e., total binding > non-specific binding). Exemplarily, the MRM chromatograms characterizing total binding (full line) and non-specific binding (dashed line) of the components from sublibrary A obtained in this way are shown in Figure 2B. In the corresponding enlarged sections below, specific binding—as difference between total and nonspecific binding—for the GAT1 inhibitors DDPM2565 (Figure 2C) and DDPM2330 (Figure 2D) is clearly visible. In contrast, compounds not binding at the NO711 binding site of GAT1 show almost the same peak intensities in both chromatograms as exemplified for meclozine (Figure 2E).

To compensate for the slight variations in the compounds' peaks intensities in the obtained MRM chromatograms caused by the LC-ESI-MS system, we did not directly compare the peak areas for hit identification but the corresponding normalized peak areas calculated as the quotient of the individual library component area vs. the internal standard [^2^H_10_]NO711 area, as shown in Table 1 exemplarily for sublibrary A (the results for the other sublibraries are compiled in Table S7). But even then, i.e., when normalized peak areas are used, total binding higher than non-specific binding may be observed for individual library components which are not due to specific binding at GAT1, but are random results due to inevitable scattering of the data obtained for the triplicate samples, going back to the binding experiments and the LC-MS quantification. Indeed, in sublibrary A we observed distinctly higher normalized areas for total binding as compared to non-specific binding as expected for the GAT1 inhibitors DDPM2565 (p*K*_i_ 7.83) and DDPM2330 (p*K*_i_ 7.15), but also slightly higher ones for chlorpromazine, doxepin, oxazepam, and telmisartan, compounds not characterized as GAT1 inhibitors so far (see Table 1).

**Table 1:** Hit identification for sublibrary A.

**Compound**	**First experiment**		**Second experiment**		**Third experiment**	
	**Total binding (normalized area)**	**Non-specific binding (normalized area)**	**Total binding (normalized area)**	**Non-specific binding (normalized area)**	**Total binding (normalized area)**	**Non-specific binding (normalized area)**
4-(4-chlorophenyl)-piperidin-4-ol	n.d	n.d	n.d	n.d	n.d	n.d
8-OH DAPT	0.177 ± 0.006	0.193 ± 0.004	0.303 ± 0.012	0.283 ± 0.042	0.176 ± 0.012	0.162 ± 0.027
Chlorpromazine	6.590 ± 0.156	6.370 ± 0.198	6.173 ± 0.693	5.663 ± 0.597	6.147 ± 0.314	6.280 ± 0.928
DDPM2330	0.771 ± 0.003	0.738 ± 0.002	0.876 ± 0.048	0.813 ± 0.031	0.490 ± 0.038	0.368 ± 0.021
DDPM2565	0.571 ± 0.040	0.119 ± 0.006	0.799 ± 0.063	0.335 ± 0.011	0.454 ± 0.012	0.069 ± 0.017
Doxepin	0.705 ± 0.025	0.666 ± 0.008	0.932 ± 0.064	0.827 ± 0.096	0.433 ± 0.056	0.404 ± 0.084
Fenoterol	0.041 ± 0.008	0.041 ± 0.002	0.034 ± 0.002	0.030 ± 0.004	0.021 ± 0.003	0.021 ± 0.002
Ketoprofen	0.004 ± 0.000	0.005 ± 0.000	0.003 ± 0.000	0.003 ± 0.000	0.004 ± 0.000	0.004 ± 0.001
Meclozine	4.937 ± 0.861	6.095 ± 0.205	5.307 ± 0.931	5.253 ± 1.007	5.188 ± 0.271	5.523 ± 0.934
Metoclopramide	0.370 ± 0.013	0.390 ± 0.028	0.503 ± 0.027	0.457 ± 0.054	0.317 ± 0.017	0.281 ± 0.042
Oxazepam	0.132 ± 0.001	0.130 ± 0.026	0.120 ± 0.020	0.111 ± 0.007	0.097 ± 0.014	0.106 ± 0.024
Piroxicam	0.054 ± 0.000	0.060 ± 0.005	0.034 ± 0.001	0.034 ± 0.003	0.040 ± 0.001	0.037 ± 0.004
Procaine	0.073 ± 0.003	0.074 ± 0.006	0.092 ± 0.003	0.082 ± 0.010	0.073 ± 0.005	0.063 ± 0.008
Roxithromycin	0.020 ± 0.003	0.021 ± 0.001	0.003 ± 0.001	0.003 ± 0.001	0.012 ± 0.001	0.010 ± 0.001
Sulpiride	0.054 ± 0.002	0.056 ± 0.004	0.068 ± 0.002	0.061 ± 0.009	0.055 ± 0.003	0.047 ± 0.003
Telmisartan	3.853 ± 0.130	3.405 ± 0.106	2.577 ± 0.326	2.290 ± 0.340	2.257 ± 0.122	2.183 ± 0.455

*n.d., not determined*.

*Total and nonspecific binding of each sublibrary component at a concentration of 1 μM (three experiments, each performed in triplicates, nonspecific binding determined in presence of 100 mM GABA). Reported values represent means ± SD. Values marked gray represent identified hits based on significantly higher total than nonspecific binding (one-tailed t-test, CL = 97.5%)*.

To investigate the probability of potential false positive results due to such random effects, we exemplarily repeated the complete screening procedure two times for sublibrary A. The results obtained in the affinity selection step for hit identification are shown in Table 1. For the GAT1 inhibitors DDPM2565 and DDPM2330, the normalized peak areas for total binding were in both repetitions again distinctly higher than those for non-specific binding. For the other compounds with normalized areas for total binding slightly exceeding those for non-specific binding in the first experiment, however, the results were not strictly consistent. Only for telmisartan, the determined total binding was higher in all experiments, whereas for chlorpromazine, doxepin, and oxazepam, this was only the case in one or two experiments. Furthermore, four additional compounds (8-OH DPAT, procaine, metoclopramide, and sulpiride) showing a similar behavior (i.e., higher total binding only in one or two experiments) were found in these repetition experiments (see Table 1). Considering these results, we decided to implement a simple statistical criterion to exclude as far as possible false positive results caused by random effects. Therefore, we performed a one-tailed *t*-test to identify those compounds with a total binding significantly exceeding non-specific binding and found that this aim could be fairly well-achieved when the confidence level was set at 97.5%. Applying this criterion to sublibrary A, both GAT1 inhibitors DDPM2565 and DDPM2330 were categorized as hits in all three experiments, whereas telmisartan and sulpiride did only show significant specific binding in one of three experiments. The results for the other seven sublibraries (B-H) obtained in this way are compiled in Table S7 and can be summarized as following. Affinity selection analyzed by LC-MS as described above led to identification of all known GAT1 inhibitors in sublibraries A-F (DDPM1349, DDPM1981, DDPM2009, DDPM2029, DDPM2077, DDPM2187, DDPM2188, DDPM2330, DDPM2473, DDPM2565, and DDPM3138) as hits. At this point, it is worth mentioning that the GAT1 inhibitor DDPM2077 with a p*K*_i_ of 5.94 could be also found as hit, leading to the conclusion, that it is indeed possible to identify ligands up to an affinity of about 1 μM under the chosen screening conditions. In contrast, the non-selective and very weak GAT inhibitor DDPM3139 with a p*K*_i_ of 4.18 at GAT1, was not identified as hit. Last but not least, it also worth mentioning, that in sublibraries containing two GAT1 inhibitors always both could be identified. Additionally, to the known GAT1 inhibitors, two further compounds namely, pilocarpine (sublibrary B) and trifluoperazine (sublibrary G) were classified as hits. The obtained normalized areas for total and non-specific binding samples for all classified hits are shown in Figure 4B. With respect to trifluperazine, however, it could already be concluded, that the affinity of this compound must be distinctly below the envisaged affinity limit (*K*_i_ of about 700 nM), as sublibrary G did not reduce NO711 binding below 50% at a concentration of 1 μM.

In addition to hit identification based on the normalized areas obtained for total vs. non-specific binding as described above, it should be examined, if the recorded data can also give indications regarding affinity of the identified hits. Therefore, the concentrations of the hits specifically bound at the NO711 binding site of GAT1 were calculated from the obtained normalized areas making use of the relative response factor (RRF, see Table S6) determined for each library component before as summarized in Table 2.

**Table 2:** Estimation of concentrations for specific binding of compounds classified as hits in the deliberately compiled 128 compound library based on RRFs.

**Sublibrary**	**GAT1 inhibitor**	**Specific binding (nM)**	**p*K_***i***_***
A	DDPM2330	0.72	7.15
	DDPM2565	10.0	7.83
	Telmisartan	1.58	n.d.
B	DDPM3138	1.90	7.81
	DDPM2188	0.38	6.42
	Pilocarpine	0.24	n.d.
C	DDPM2077	0.48	5.94
	DDPM2029	3.18	6.32
D	DDPM2187	0.22	6.50
	DDPM2473	3.44	8.13
E	DDPM1981	2.20	7.04
	DDPM1349	0.20	6.40
F	DDPM2009	2.95	6.16
G	Trifluoperazine	1.46	n.d.

*n.d., pK_i_ value unavailable*.

In this context, it is, however, important to point out that such concentrations calculated correspondingly, can only be rough estimates and do by far not have the quality of results determined according to a validated quantification by means of LC-MS. Interestingly, in all libraries with two known GAT1 inhibitors, a higher concentration of specific binding was found for the compound with the higher affinity as compared to the one with the lower affinity. Furthermore, apart from sublibrary A, the total concentrations of bound GAT1 inhibitors in the sublibraries seem to be in the range of about 2–3 nM, which is well in agreement with a GAT1 concentration of about 3 nM in the binding experiment. In contrast, the estimated concentration of 10 nM for the specific binding of DDPM2565 is way too high considering that the target concentration amounts only to ~3 nM, which as the upper limit for binding is also well-confirmed by the binding data found for the other GAT1 inhibitors (DDPM1349, DDPM1981, DDPM2009, DDPM2029, DDPM2077, DDPM2187, DDPM2188, DDPM2330, DDPM2473, and DDPM3138). A closer investigation of this phenomenon revealed strong adherence of DDPM2565 to various surfaces especially when dissolved in aqueous milieu. This results in an overestimated RRF-value and therefore in an overestimated concentration of bound compound.

In summary, it can be concluded, that the results obtained according to our established affinity selection protocol allow a rough categorization of affinity, but definitively not the establishment of a detailed affinity rank order. In this context, it should be kept in mind again, that the concentrations calculated for specific binding do not reflect equilibrium binding concentrations due to the dissociation issue during the washing process.

Finally, we demonstrated, that assessment of library activity and hit identification can also be accomplished simultaneously employing the gradient based LC method. To this end, the mass traces for NO711 together with those of the individual sublibrary components were recorded in MRM chromatograms under gradient conditions. Thereby, in a single set of LC-MS runs remaining reporter ligand binding in the presence of a sublibrary as well as total and non-specific binding of the library components could be quantified. Following this approach, the observed reduction of reporter ligand binding for sublibraries A-H was almost the same as determined before in two separate steps (see Table S8).

#### Subsequent Investigations—Activity Assessment of Reduced Sublibraries and Hit Verification

Furthermore, Additional experiments should be performed that allow to clarify whether there are further hits besides the ones already identified in sublibraries A-F (characterized as active) during the affinity selection process, which may have been missed due to insufficient sensitivity of LC-MS quantification or due to competitive effects in a sublibrary (i.e., in the presence of high-affinity ligands). This should be assessed by measuring the activities of the reduced sublibraries delineated form the active sublibraries by omitting the hits identified before in additional competitive binding experiments. The results obtained in this way are shown in Figure 4C. In contrast to the original sublibraries A-F, the corresponding reduced sublibraries did hardly reduce reporter ligand binding, indicating that there are no further GAT1 ligands with significant affinity in these sublibraries.

Finally, the activity of compounds classified as hits during the affinity selection process should be verified. As the affinities of the identified GAT1 inhibitors DDPM1349, DDPM1981, DDPM2009, DDPM2029, DDPM2077, DDPM2187, DDPM2188, DDPM2330, DDPM2473, DDPM2565, and DDPM3138 had already been assessed earlier (see Table 2), we focused on the compounds additionally classified as hits in the present study, namely telmisartan, pilocarpine, and trifluoperazine (we did not include sulpiride, as this compound was only classified as hit in the exemplarily repeated experiments for sublibrary A). Investigating the three compounds individually in competitive binding experiments showed that none of the compounds could distinctly inhibit NO711 binding as shown in Figure 4D, clearly indicating that these compounds do not have significant affinity toward the NO711 binding site of GAT1. In this sense, these compounds represent false positive hits, nevertheless, it cannot be excluded that these compounds might bind to GAT1 at another binding site which is also addressed by GABA. Corresponding investigations have to be done and are not yet finished, at least for trifluoperazine, however, inhibition of GAT1 characterized by a pIC_50_ value of 4.62 could already be found. Summing up the results of these subsequent investigations in the context of the complete screening protocol, it can be concluded, that both false positive as well as false negative hits can be reliably avoided in this way.

### Proof of Concept—Screening of the Tocrisscreen Plus Library for Ligands Addressing GAT1

**Figure 5:**
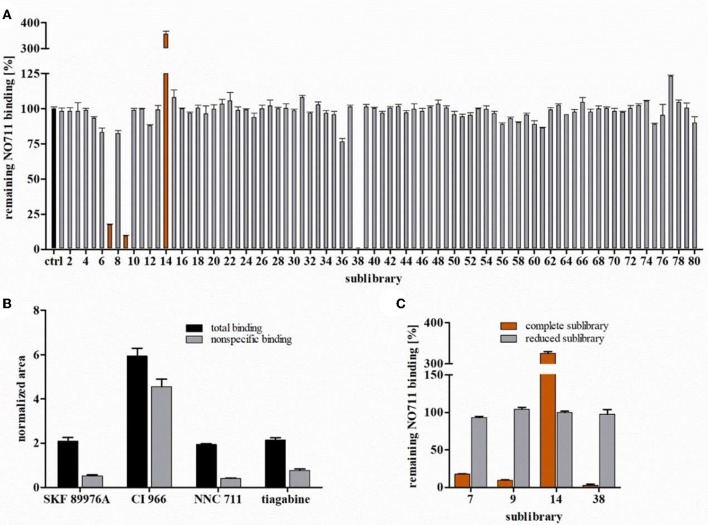
Screening of the Tocris Plus Library. **(A)** Remaining NO711 binding % in presence of the sublibraries 1–80. **(B)** Total and non-specific binding of all identified hits at a concentration each of 1 μM for each sublibrary component. Non-specific binding was determined in the presence of 100 mM GABA. **(C)** Remaining NO711 binding % determined in presence of the sublibraries and corresponding reduced sublibraries (without identified hits). Reported values represent means ± SD (*n* = 3).

As a proof of concept of the presented strategy, we applied the developed protocol to screen 1,280 well-characterized pharmacological tool compounds of the Tocris Screen Plus library for ligands addressing the NO711 binding site of GAT1. The entire library was again divided in sublibraries containing 16 compounds, but in this case the sequence set by Tocris was left unchanged or in other words the sublibraries were composed without considering the identity of the library components. This means that the sublibraries were compiled absolutely by random (i.e., without knowledge of molecular weight or biological activity of the individual compounds). Following the established screening protocol, we investigated at first the sublibraries regarding their potency to inhibit reporter ligand binding (again with the individual components at a final concentration of 1 μM). Sublibrary 7, sublibrary 9 as well as sublibrary 38 decreased reporter ligand binding to 12, 8, and 4%, respectively, i.e., distinctly under the defined 50% limit, and were therefore classified as active, whereas the 77 other sublibraries were classified inactive. Surprisingly, for one of these sublibraries, namely sublibrary 14, we determined an unexpectedly high value of 360% of remaining reporter ligand binding (Figure 5A). As artifacts due to cross contamination during the process of the binding experiment or cross talk phenomena during LC-ESI-MS/MS analysis could be excluded, we decided to subject sublibrary 14 together with sublibraries 7, 9, and 38 to the hit identification procedure. For the recording of the MRM chromatograms of all components of the active sublibraries the fragmentation (again employing the automated procedure of the mass spectrometer) of the respective constituents had to be determined. To this end, it was necessary to check the identity of the corresponding sublibrary components. Thereby, it turned out that NO711 (referred to as NNC711 in the Tocris library), which is the compound used as reporter ligand in our competitive binding experiment, is a member of sublibrary 14, for which a specific reporter ligand binding of 360% had been found. The observed high reporter ligand binding in this sublibrary is therefore quite simple to explain. It is to be attributed to an enhanced NO711 concentration of 1010 nM in comparison with 10 nM in the control (i.e., in the absence of any GAT ligands), almost completely saturating the GAT1 binding sites and consequently leading to a concentration of bound NO711 close to the total concentration of GAT1 in the binding sample. Additionally, in the other active sublibraries, we also recognized well-known GAT1 inhibitors at this step, namely SKF 89976A (sublibrary 7), CI 966 (sublibrary 9), and tiagabine (sublibrary 38). Despite this knowledge, we followed the established screening protocol to prove, that these GAT1 inhibitors and possibly also other members of the library can be identified as hits.

Initially, we examined again the MRM chromatograms obtained for matrix samples containing the library components of sublibraries 7, 9, 14, and 38 in a concentration of 1 nM, based on the mass transitions generated with the automatic optimization tool of the mass spectrometer. These chromatograms revealed that all compounds except for DuP 697 (sublibrary 9), IEM 1460 (sublibrary 14), and flurizan (sublibrary 38) could be quantified with sufficient sensitivity under these conditions. This means that the rate of quantifiable compounds is again about 95% (detailed results obtained from these MRM chromatograms are shown in the Table S9). Next, we recorded MRM chromatograms for the samples representing total and non-specific binding, respectively, of sublibraries 7, 9, 14, and 38 (see Table S10). Out of all library components investigated in this way, only SKF 89976A (sublibrary 7), CI 966 (sublibrary 9), NNC711 (sublibrary 14), and tiagabine (sublibrary 38), revealed significantly higher total than non-specific binding (based on the corresponding normalized areas) as shown in Figure 5B and could thus be classified as hits. Accordingly, again all “active” ligands could be unveiled by a significant specific binding to the NO711 labeled binding site of GAT1 under the applied conditions and unambiguously identified as hits by our screening concept. The concentrations calculated for specific binding of these GAT1 inhibitors (based on the corresponding RRFs) are shown in Table 3. The conclusions to be drawn from these results are again, that the concentrations determined for high-affinity ligands reflecting their specific binding are close to the target concentration and furthermore, that the affinities of SKF 89976A, CI 966, and tiagabine can be estimated to be roughly in the same order of magnitude as NO711.

**Table 3:** Estimation of the concentration of specifically bound GAT1 inhibitors of the Tocris Screen Plus library based on RRFs.

**Sublibrary**	**GAT1 inhibitor**	**Specific binding (nM)**
7	SKF 89976A	2.24
9	CI 966	3.04
14	NNC711 (NO711)	2.93
38	Tiagabine	3.28

Finally, we removed all identified hits (i.e., SKF 89976A from sublibrary 7, CI 966 from sublibrary 9, NNC711 from sublibrary 14, and tiagabine from sublibrary 38) and studied the resulting “reduced sublibraries” in a further competitive binding experiment for inhibition of reporter ligand binding. The percentages of remaining bound reporter ligand determined for the reduced sublibraries 7 (93%), 9 (104%), 14 (100%), and 38 (97%) are depicted in Figure 5C (together with the percentages obtained before for the complete sublibraries). Thereby, it could be demonstrated that these sublibraries do not contain further ligands with significant affinity for the NO711 binding site of GAT1, beyond the above mentioned known GAT1 inhibitors.

As stated in the section “Basic considerations before implementation of the library screening concept,” the sublibrary size is flexible and not limited. It can easily be increased to more than 16 constituents, the so far used maximum number. To demonstrate this, the former sublibraries 5-8 were pooled to give a sublibrary comprising 64 compounds. For this 64 compound sublibrary, containing the GAT1 inhibitor SKF 89976A, a reduction of reporter ligand binding down to 3% was determined in the first step. For hit identification, in this case, the mass transitions of 64 components had to be investigated (from which the ones for sublibrary 7 were already known). Again three out of 64 compounds, namely brefeldin A, olvanil, and SDZ 220-581 could not be quantified based on the automatically generated mass transitions in the resulting MRM chromatograms, the success rate for quantifiable compounds down to a concentration of at least 1 nM was again amounting to 95% (Table S9). Then in analogy to the procedure described above for the sublibraries containing 16 components, total and non-specific binding were determined for the 64 constituents comprising libraries (for detailed results see Table S11). As expected, only SKF 89976A gave a significant specific binding and this could be identified as hit.

The results obtained here demonstrate that our strategy to combine MS Binding Assays and ASMS is indeed successful to overcome the weaknesses of both methods. While screening of the 1,280 compound library by MS Binding Assays alone would require elaborate deconvolution of the four active sublibraries, sole screening on basis of ASMS would come along with great efforts for investigation of 1,280 compounds' mass transitions. In contrast, the combination of both concepts reduces these efforts distinctly, as only 64 of 1,280 compounds have to be characterized for their mass transitions. Additionally, the efforts for deconvolution of four sublibaries can be avoided. Last but not least should be mentioned, that even ligands with weak MS sensitivities, which would stay unidentified in conventional ASMS approaches, can be reliably found.

## Conclusion

In the present study, the concepts of competitive MS Binding Assays and ASMS were combined to a new, powerful, efficient, and reliable library screening approach. It starts with a filtration based competitive binding experiment, that is in the first step analyzed for reduced binding of the reporter ligand, and—only if a sublibrary is active—additionally for individual library components showing specific binding (i.e., total binding surpassing non-specific binding). In this way, the strengths of MS Binding Assays and ASMS, i.e., the unambiguous indication of the presence or absence of hits in a library by MS Binding Assays and the efficiency of hit identification by ASMS are merged. Correspondingly, also the weaknesses of both concepts, i.e., the time consuming deconvolution strategies for hit identification in MS Binding Assays and the issue of false negative or false positive results inherently coupled with ASMS, can be avoided. Application of this concept to screening of a small, deliberately compiled library of 128 compounds and a medium-size library of 1,280 compounds for ligands addressing the neuronal GABA transporter GAT1 demonstrated its capability to identify all hits present in the libraries down to an affinity characterized by a p*K*_*i*_ value of about 6.

The concept is based on a high-sensitive LC-ESI-MS method employing a triple quadrupole mass spectrometer (without being restricted to this instrument type) for quantification of a broad variety of small molecule compounds down to the pM concentration level. In this way, binding of 95% of all investigated test compounds could be quantified, distinctly surpassing the success rates for mass spectrometric detection of common ASMS approaches. This highly sensitive quantification enables very low target concentrations (in the low nM range) in the binding experiment, rendering the screening concept particularly attractive for membrane integrated drug targets. Therefore, it does, in contrast to ASMS approaches, not demand high sophisticated expression systems and purification methods for a target of interest. But, to allow reliable hit identification, quantification of library components down to an LLOQ distinctly below the employed target concentration should be possible. With respect to the number of compounds investigated in a single binding experiment (here referred to as “sublibrary”), the concept is very flexible and can be adopted at will depending e.g., on the expected hit rate, the defined activity criterion, or the size of the entire library. In this study, 16 membered sublibraries proved to be very efficient as well as a 64 membered sublibrary investigated exemplarily, but even higher numbers can be envisaged as long as all the compounds can be quantified in a single LC-MS run.

As the presented strategy defines “activity” of compounds in competitive MS Binding Assays, it is ideally suited for the screening of compounds addressing a distinct binding site at a target—that is the one addressed by the reporter ligand. However, the presented concept is not restricted to screening toward ligands occupying this binding site. Inhibition of reporter ligand binding, not due to competitive interactions and furthermore, compounds enhancing reporter ligand binding can be detected as well. It has to be kept in mind only at this point, that non-specific binding of library components has to be defined appropriately to achieve this goal, for example employing a membrane preparation (or another suitable source) lacking the target instead of adding a competitive ligand in high excess, as it was done in this study. Considering the capabilities and the potential provided by the combination of competitive MS Binding Assays and ASMS, the concept described here can be assumed to become a valuable and powerful tool in early drug research.

## Data Availability Statement

All datasets generated for this study are included in the manuscript/Supplementary Files.

## Author Contributions

JG, GH, and KW wrote or contributed to the writing of the manuscript and participated in research study design. JG carried out laboratory and data analysis.

### Dedication

This article is dedicated to Prof. Dr. Dr. h.c. mult. Hildebert Wagner with warmest wishes on the occasion of his 90th birthday.

### Conflict of Interest

The authors declare that the research was conducted in the absence of any commercial or financial relationships that could be construed as a potential conflict of interest.
