# Supplementary material for: A Library Screening Strategy Combining the Concepts of MS Binding Assays and Affinity Selection Mass Spectrometry
Source: Front Chem. 2019 Oct 4;7:665. doi: 10.3389/fchem.2019.00665 (PMC6787468; doi:10.3389/fchem.2019.00665)
Supplement: Supplementary file 1 [file Table_1.DOCX]

**A library screening strategy combining the concepts of MS Binding Assays and affinity selection mass spectrometry**

Supplementary Material

**Content**

**LC-ESI-MS/MS method development**

**Table S1**: GAT1 inhibitors of the deliberately compiled library.

**Table S2**: Software settings for automated determination of compound-dependent MS parameters.

**Table S3**: Compound-dependent MS parameters for components of the deliberately compiled library.

**Table S4**: Compound-dependent MS parameters for components of the Tocris Screen Plus library.

**Table S5**: IC_50_ to p*K_i_* transformation and calculations of the equilibrium binding concentrations.

**Table S6**: Results obtained for components of the deliberately compiled library from MRM chromatograms.

**Table S7**: Hit identification for the deliberately compiled library.

**Table S8**: Remaining NO711 binding determined by means of gradient LC-ESI-MS/MS.

**Table S9**: Results obtained for components of the Tocris Screen Plus library from MRM chromatograms.

**Table S10**: Hit identification of Tocris Screen Plus library (16 membered sublibraries).

**Table S11**: Hit identification of Tocris Screen Plus library (64 membered sublibrary).

**LC-ESI-MS/MS method development**

We included all eight sublibraries in this step in order to develop a “universal” HPLC method suitable for compounds reflecting a broad spectrum of chemical diversity. The already established isocratic LC-ESI-MS/MS method for quantification of the reporter ligand NO711, based on a C18 stationary phase in combination with 10 mM ammonium formate buffer at pH 7.0 and acetonitrile (50/50, v/v, for details see experimental section), was assumed to be not suitable for this purpose, as the polar components of the library would elute with the void volume, whereas the highly lipophilic ones would be retained extremely long on the stationary phase. In order to overcome these problems, a gradient elution LC method was developed. To keep LC-MS analysis as simple as possible and due to our experience with the chromatographic behavior of matrix generated in MS Binding Assays, we decided to employ the same C18 stationary phase and again 10 mM ammonium formate buffer at pH 7.0 together with acetonitrile as mobile phase as used before for our MS Binding Assay. We found the following gradient conditions appropriate for our purpose: For the start of the chromatography, a solvent mixture with a ratio of 60/40 (v/v, 10 mM ammonium formate, pH 7.0 / acetonitrile) had to be used, which after 0.01 min had to be rapidly changed to a solvent ratio of 20/80 thus reducing the aqueous component. This solvent ratio remained unchanged until the run time amounted to 3.5 min. Then, the solvent ratio was rapidly switched back to original conditions (60/40 v/v). This solvent ratio was applied for 5.5 min thus leading to a total runtime of 9.0 min for each sample injection.

**Table S1:** GAT1 inhibitors of the deliberately compiled library. All p*K_i_* values were assessed by in house competitive MS Binding Assays.

| **DDPM1349**  (p*K*_i_ 6.40)  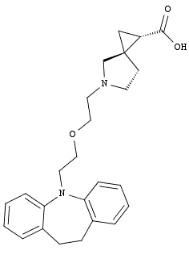 | **DDPM1981**  (p*K*_i_ 7.04)  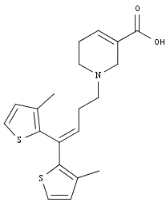 | **DDPM2009**  (p*K*_i_ 6.16)  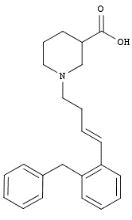 | **DDPM2029**  (p*K*_i_ 6.32)  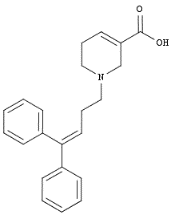 | **DDPM2077**  (p*K*_i_ 5.94)  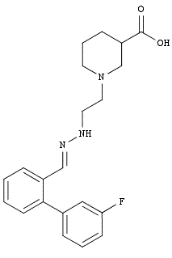 |
| --- | --- | --- | --- | --- |
| **DDPM2187**  (p*K*_i_ 6.50)  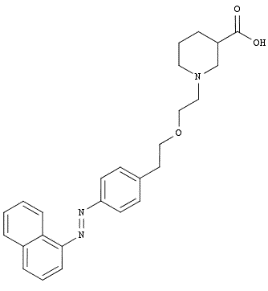 | **DDPM2188**  (p*K*_i_ 6.42)  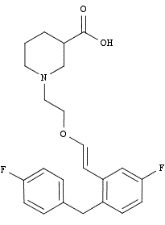 | **DDPM2330**  (p*K*_i_ 7.15)  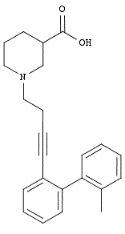 | **DDPM2473**  (p*K*_i_ 8.13)  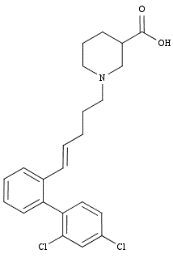 | **DDPM2565**  (p*K*_i_ 7.83)  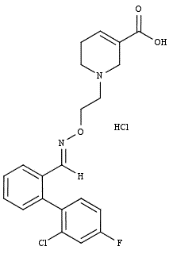 |
| **DDPM3138**  (p*K*_i_ 7.81)  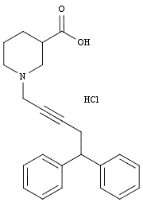 | **DDPM3139**  (p*K*_i_ 4.18)  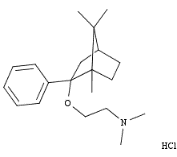 |  |  |  |

**Table S2:** Software settings for automated determination of compound-dependent MS parameters.

| polarity | pos /neg |
| --- | --- |
| **precursor ion** |  |
| resolution: | unit |
| search window: | +/- 0.500 Da |
| **product Ion** |  |
| resolution: | unit |
| from the most intense: | 8 peaks |
| build final method using : | 8 most intensive peaks |
| exclude Product ion: | +/- 5.000 Da |
| min. mass for Product ion: | 50.000 Da |
| threshold for Product Ion: | 0.000 cps |

**Table S3:** Compound-dependent MS parameters for components of the deliberately compiled library.

| **compound** | **ESI** | | **Q1**  **(Da)** | **Q3**  **(Da)** | **DP**  **(V)** | **EP**  **(V)** | **CE**  **(V)** | **DXP**  **(V)** |
| --- | --- | --- | --- | --- | --- | --- | --- | --- |
| 2-(4-methyl-1,4-diazepan-1-yl)benzoic acid | | + | 235 | 58 | 61 | 10 | 25 | 8 |
| 4-(4-chloro-phenyl)-piperidin-4-ol | | + | 212 | 194 | 51 | 10 | 13 | 20 |
| 8-OH DAPT | | + | 248 | 147 | 81 | 10 | 25 | 16 |
| aciclovir | | + | 226 | 198 | 231 | 10 | 35 | 22 |
| ambroxol | | + | 377 | 262 | 46 | 10 | 23 | 28 |
| amitriptyline | | + | 278 | 233 | 111 | 10 | 23 | 20 |
| antazoline | | + | 266 | 91 | 101 | 10 | 33 | 10 |
| ASP+ | | + | 240 | 224 | 120 | 10 | 41 | 20 |
| atenolol | | + | 267 | 145 | 136 | 10 | 35 | 16 |
| atropine | | + | 290 | 93 | 156 | 10 | 37 | 10 |
| baclofen | | + | 214 | 151 | 51 | 10 | 25 | 16 |
| benzylpenicillin | | + | 335 | 176 | 50 | 10 | 28 | 16 |
| bifonazole | | + | 311 | 243 | 50 | 10 | 20 | 24 |
| biperiden | | + | 312 | 98 | 81 | 10 | 29 | 12 |
| brucine | | + | 395 | 244 | 216 | 10 | 49 | 22 |
| buspirione | | + | 240 | 131 | 56 | 10 | 35 | 14 |
| cetirizine | | + | 389 | 201 | 81 | 10 | 23 | 18 |
| quinine | | + | 325 | 160 | 56 | 10 | 39 | 16 |
| clonidine | | + | 230 | 109 | 166 | 10 | 63 | 10 |
| chlorpromazine | | + | 319 | 58 | 91 | 10 | 65 | 8 |
| chlordiazepoxide | | + | 300 | 283 | 56 | 10 | 31 | 22 |
| chloroquine | | + | 320 | 142 | 66 | 10 | 29 | 16 |
| cimetidine | | + | 253 | 159 | 61 | 10 | 19 | 14 |
| ciprofloxacin | | + | 332 | 314 | 111 | 10 | 27 | 28 |
| clomipramine | | + | 315 | 242 | 101 | 10 | 33 | 18 |
| clotrimazole | | + | 345 | 328 | 71 | 10 | 13 | 30 |
| diazepam | | + | 285 | 193 | 146 | 10 | 43 | 22 |
| diphenhydramine | | + | 256 | 167 | 81 | 10 | 13 | 10 |
| diltiazem | | + | 415 | 178 | 146 | 10 | 31 | 18 |
| doxepin | | + | 280 | 107 | 106 | 10 | 29 | 12 |
| drofenine | | + | 318 | 100 | 86 | 10 | 25 | 12 |
| DTG | | + | 240 | 108 | 126 | 10 | 29 | 12 |
| ethacridine | | + | 254 | 197 | 146 | 10 | 43 | 20 |
| fenoterol | | + | 304 | 107 | 96 | 10 | 35 | 12 |
| DDPM1349 | | + | 407 | 168 | 106 | 10 | 33 | 16 |
| DDPM1981 | | + | 374 | 247 | 126 | 10 | 23 | 20 |
| DDPM2009 | | + | 350 | 179 | 111 | 10 | 37 | 16 |
| DDPM2029 | | + | 334 | 129 | 56 | 10 | 29 | 14 |
| DDPM2077 | | + | 370 | 156 | 141 | 10 | 31 | 14 |
| DDPM2187 | | + | 432 | 156 | 101 | 10 | 31 | 16 |
| DDPM2188 | | + | 402 | 156 | 191 | 10 | 33 | 16 |
| DDPM2330 | | + | 418 | 191 | 181 | 10 | 69 | 18 |
| DDPM2473 | | + | 348 | 202 | 121 | 10 | 93 | 20 |
| DDPM2565 | | + | 403 | 154 | 110 | 10 | 30 | 10 |
| DDPM3139 | | + | 302 | 213 | 41 | 10 | 15 | 20 |
| **Table S3 (continued)** | |  |  |  |  |  |  |  |
| **compound** | **ESI** | | **Q1**  **(Da)** | **Q3**  **(Da)** | **DP**  **(V)** | **EP**  **(V)** | **CE**  **(V)** | **DXP**  **(V)** |
| DDPM3138 | | + | 348 | 167 | 176 | 10 | 39 | 18 |
| glibenclamide | | + | 494 | 369 | 111 | 10 | 19 | 28 |
| haloperidol | | + | 376 | 165 | 66 | 10 | 31 | 16 |
| hydromorphone | | + | 286 | 185 | 136 | 10 | 39 | 14 |
| imipramine | | + | 281 | 86 | 86 | 10 | 21 | 10 |
| indometacin | | + | 358 | 139 | 66 | 10 | 23 | 14 |
| ipratropium | | + | 333 | 166 | 121 | 10 | 35 | 14 |
| isoprenaline | | + | 212 | 100 | 96 | 10 | 37 | 18 |
| ketoprofen | | + | 255 | 105 | 126 | 10 | 29 | 12 |
| lidocaine | | + | 235 | 86 | 81 | 10 | 21 | 10 |
| lisinopril | | + | 406 | 84 | 71 | 10 | 29 | 8 |
| meclozine | | + | 391 | 201 | 96 | 10 | 21 | 18 |
| meloxicam | | + | 352 | 73 | 111 | 10 | 77 | 10 |
| mepivacaine | | + | 247 | 98 | 106 | 10 | 23 | 12 |
| meprobamate | | + | 219 | 158 | 61 | 10 | 11 | 14 |
| metipranolol | | + | 310 | 191 | 81 | 10 | 29 | 16 |
| metoclopramide | | + | 300 | 227 | 61 | 10 | 25 | 20 |
| metoprolol | | + | 268 | 116 | 111 | 10 | 25 | 10 |
| molsidomine | | + | 243 | 86 | 71 | 10 | 13 | 10 |
| morphine | | + | 286 | 152 | 136 | 10 | 75 | 10 |
| (2-morpholin-4-ylmethyl-benzoimidazol-1-yl)-acetic acid | | + | 276 | 100 | 91 | 10 | 25 | 12 |
| moxifloxacin | | + | 402 | 384 | 91 | 10 | 31 | 32 |
| nalidixic acid | | + | 233 | 215 | 61 | 10 | 17 | 18 |
| naphazoline | | + | 211 | 115 | 136 | 10 | 63 | 14 |
| noscapine | | + | 414 | 220 | 171 | 10 | 29 | 18 |
| oxazepam | | + | 287 | 241 | 111 | 10 | 29 | 22 |
| ofloxacin | | + | 362 | 318 | 71 | 10 | 25 | 26 |
| papaverine | | + | 340 | 202 | 156 | 10 | 35 | 16 |
| PH014034 | | + | 491 | 112 | 146 | 10 | 37 | 14 |
| phenobarbital | | + | 233 | 146 | 106 | 10 | 29 | 10 |
| phenylbutazone | | + | 309 | 160 | 116 | 10 | 29 | 16 |
| physostigmine | | + | 276 | 162 | 66 | 10 | 27 | 16 |
| pilocarpine | | + | 209 | 96 | 151 | 10 | 35 | 10 |
| pimozide | | + | 462 | 328 | 71 | 10 | 39 | 30 |
| piretanide | | + | 363 | 282 | 76 | 10 | 29 | 26 |
| piroxicam | | + | 332 | 95 | 71 | 10 | 25 | 12 |
| procaine | | + | 237 | 100 | 41 | 10 | 19 | 12 |
| procainamide | | + | 236 | 163 | 56 | 10 | 21 | 16 |
| promethazine | | + | 285 | 86 | 91 | 10 | 21 | 10 |
| prophyphenazone | | + | 231 | 56 | 156 | 10 | 57 | 8 |
| propranolol | | + | 260 | 116 | 111 | 10 | 23 | 12 |
| ramipril | | + | 417 | 234 | 86 | 10 | 27 | 22 |
| ranitidine | | + | 315 | 176 | 61 | 10 | 23 | 14 |
| reserpine | | + | 609 | 195 | 60 | 10 | 55 | 28 |
| riboflavin | | + | 377 | 243 | 66 | 10 | 33 | 20 |
| roxithromycin | | + | 837 | 158 | 151 | 10 | 41 | 14 |
| salbutamol | | + | 240 | 148 | 56 | 10 | 23 | 14 |
| **Table S3 (continued)** | |  |  |  |  |  |  |  |
| **compound** | **ESI** | | **Q1**  **(Da)** | **Q3**  **(Da)** | **DP**  **(V)** | **EP**  **(V)** | **CE**  **(V)** | **DXP**  **(V)** |
| scoplamine | | + | 304 | 138 | 116 | 10 | 29 | 14 |
| sertraline | | + | 306 | 275 | 61 | 10 | 15 | 24 |
| strychnine | | + | 335 | 184 | 161 | 10 | 49 | 20 |
| sulfaguanidine | | + | 215 | 156 | 81 | 10 | 19 | 16 |
| sulfamethoxazole | | + | 254 | 92 | 131 | 10 | 31 | 10 |
| sulfisomidine | | + | 279 | 124 | 146 | 10 | 27 | 14 |
| sulpiride | | + | 342 | 214 | 91 | 10 | 41 | 22 |
| telmisartan | | + | 515 | 276 | 186 | 10 | 63 | 28 |
| terfenadine | | + | 472 | 436 | 186 | 10 | 33 | 24 |
| tetracaine | | + | 265 | 176 | 66 | 10 | 19 | 16 |
| tetracycline | | + | 445 | 427 | 76 | 10 | 17 | 14 |
| tiabendazole | | + | 202 | 175 | 41 | 10 | 35 | 18 |
| tianeptine | | + | 437 | 292 | 81 | 10 | 21 | 24 |
| tolbutamide | | + | 271 | 74 | 76 | 10 | 17 | 10 |
| tramadol | | + | 264 | 58 | 56 | 10 | 53 | 8 |
| trifluoperazine | | + | 408 | 141 | 76 | 10 | 31 | 16 |
| trimethoprim | | + | 291 | 261 | 106 | 10 | 33 | 24 |
| triphenylamine | | + | 242 | 142 | 91 | 10 | 33 | 16 |
| verapamil | | + | 455 | 303 | 131 | 10 | 33 | 28 |
| xylometazoline | | + | 245 | 189 | 166 | 10 | 35 | 20 |
| 3-(2-methyl-1H-imidazol-1-yl)benzoic acid | | - | 201 | 81 | -30 | -10 | -30 | -9 |
| acetazolamide | | - | 221 | 83 | -65 | -10 | -22 | -11 |
| captopril | | - | 217 | 182 | -45 | -10 | -16 | -15 |
| chloramphenicol | | - | 321 | 152 | -50 | -10 | -24 | -19 |
| chlortalidone | | - | 337 | 190 | -115 | -10 | -22 | -23 |
| diclofenac | | - | 294 | 250 | -80 | -10 | -14 | -15 |
| etacrynic acid | | - | 301 | 243 | -40 | -10 | -14 | -19 |
| furosemide | | - | 329 | 205 | -50 | -10 | -28 | -27 |
| hydrochlorothiazide | | - | 296 | 205 | -85 | -10 | -32 | -23 |
| mefenamic acid | | - | 240 | 196 | -45 | -10 | -22 | -11 |
| methyl orange | | - | 304 | 156 | -45 | -10 | -36 | -17 |
| naproxen | | - | 229 | 169 | -45 | -10 | -36 | -19 |
| lauryl maltoside | | - | 509 | 89 | -135 | -10 | -28 | -11 |
| niclosamide | | - | 325 | 171 | -70 | -10 | -34 | -19 |
| nitrazepam | | - | 280 | 252 | -65 | -10 | -24 | -29 |
| phenytoin | | - | 251 | 102 | -20 | -10 | -32 | -25 |

**Table S4:** Compound-dependent MS parameters for components of the Tocris Screen Plus library.

| **compound** | **ESI** | **Q1**  **(Da)** | **Q3**  **(Da)** | **DP**  **(V)** | **EP**  **(V)** | **CE**  **(V)** | **CXP**  **(V)** |
| --- | --- | --- | --- | --- | --- | --- | --- |
| (-)-quinpirole | + | 220 | 161 | 146 | 10 | 27 | 14 |
| (-)-U-50488 | + | 369 | 112 | 121 | 10 | 41 | 12 |
| (+)-MK 801 | + | 222 | 205 | 126 | 10 | 23 | 18 |
| (R)-(-)-rolipram | + | 276 | 208 | 141 | 10 | 21 | 18 |
| 3'-fluorobenzylspiperone | + | 504 | 165 | 171 | 10 | 39 | 16 |
| 4E1RCat | + | 479 | 241 | 201 | 10 | 57 | 20 |
| 4-P-PDOT | + | 280 | 91 | 116 | 10 | 29 | 10 |
| 8-M-PDOT | + | 234 | 161 | 76 | 10 | 21 | 14 |
| A 582941 | + | 281 | 157 | 176 | 10 | 37 | 14 |
| ADX 10059 | + | 241 | 226 | 206 | 10 | 37 | 18 |
| AF-DX 116 | + | 422 | 349 | 106 | 10 | 29 | 30 |
| apoptosis activator 2 | + | 306 | 159 | 66 | 10 | 23 | 14 |
| AT 1015 | + | 456 | 183 | 86 | 10 | 39 | 14 |
| BD 1047 | + | 275 | 230 | 56 | 10 | 21 | 20 |
| BIMU 8 | + | 343 | 167 | 136 | 10 | 29 | 14 |
| BNTX | + | 430 | 267 | 81 | 10 | 35 | 22 |
| BW 723C86 | + | 287 | 97 | 71 | 10 | 27 | 12 |
| calhex 231 | + | 407 | 155 | 91 | 10 | 27 | 14 |
| castanospermine | + | 190 | 172 | 51 | 10 | 19 | 14 |
| CGP 54626 | + | 408 | 236 | 51 | 10 | 25 | 20 |
| CGP 55845 | + | 402 | 230 | 71 | 10 | 23 | 18 |
| CGP 7930 | + | 293 | 72 | 86 | 10 | 19 | 6 |
| CI 966 | + | 474 | 303 | 86 | 10 | 29 | 26 |
| cilostamide | + | 343 | 182 | 116 | 10 | 21 | 16 |
| cisapride | + | 466 | 184 | 81 | 10 | 37 | 16 |
| clemastine | + | 344 | 180 | 71 | 10 | 43 | 16 |
| CMPD-1 | + | 350 | 241 | 171 | 10 | 25 | 20 |
| CP 94253 | + | 258 | 187 | 86 | 10 | 29 | 18 |
| CPCCOEt | + | 248 | 157 | 41 | 10 | 25 | 14 |
| daidzein | + | 255 | 152 | 136 | 10 | 53 | 16 |
| DAU 5884 | + | 316 | 124 | 111 | 10 | 33 | 12 |
| DBeQ | + | 341 | 250 | 171 | 10 | 33 | 20 |
| DCEBIO | + | 231 | 203 | 116 | 10 | 29 | 22 |
| DH 97 | + | 335 | 234 | 91 | 10 | 23 | 18 |
| dibutyryl-cAMP | + | 470 | 206 | 86 | 10 | 15 | 18 |
| EMD 281014 | + | 377 | 114 | 16 | 10 | 31 | 12 |
| FH 535 | + | 359 | 145 | 80 | 10 | 36 | 21 |
| flecainide | + | 415 | 98 | 171 | 10 | 33 | 12 |
| formoterol | + | 345 | 149 | 40 | 10 | 25 | 16 |
| FPL 64176 | + | 348 | 91 | 86 | 10 | 23 | 8 |
| FR 139317 | + | 605 | 367 | 101 | 10 | 27 | 10 |
| GBR 12909 | + | 451 | 181 | 141 | 10 | 129 | 16 |
| GR 159897 | + | 415 | 110 | 166 | 10 | 33 | 10 |
| GYKI 52466 | + | 294 | 160 | 146 | 10 | 41 | 18 |
| **Table S4 (continued)** |  |  |  |  |  |  |  |
| **compound** | **ESI** | **Q1**  **(Da)** | **Q3**  **(Da)** | **DP**  **(V)** | **EP**  **(V)** | **CE**  **(V)** | **CXP**  **(V)** |
| ICA 069673 | + | 270 | 113 | 56 | 10 | 57 | 10 |
| ICA 069673 | + | 268 | 73 | 65 | 10 | 82 | 9 |
| KB-R7943 | + | 332 | 256 | 111 | 10 | 21 | 22 |
| KU14R | + | 215 | 137 | 146 | 10 | 25 | 14 |
| L-655,240 | + | 374 | 125 | 61 | 10 | 27 | 10 |
| L-655,708 | + | 342 | 296 | 81 | 10 | 23 | 24 |
| L-701,324 | + | 364 | 253 | 56 | 10 | 49 | 24 |
| L-732,138 | + | 473 | 201 | 131 | 10 | 25 | 18 |
| LY 225910 | + | 502 | 295 | 156 | 10 | 35 | 26 |
| MDL 11,939 | + | 296 | 105 | 60 | 10 | 37 | 10 |
| MDL 72222 | + | 314 | 124 | 61 | 10 | 31 | 12 |
| methiothepin | + | 357 | 210 | 61 | 10 | 45 | 18 |
| methyllycaconitine | + | 683 | 216 | 101 | 10 | 63 | 18 |
| MG 624 | + | 324 | 100 | 130 | 10 | 46 | 18 |
| mirtazapine | + | 266 | 195 | 156 | 10 | 33 | 16 |
| ML 218 | + | 369 | 109 | 181 | 10 | 99 | 12 |
| MPEP | + | 194 | 92 | 166 | 10 | 35 | 10 |
| MRS 1220 | + | 404 | 286 | 91 | 10 | 25 | 24 |
| naloxone benzoylhydrazone | + | 446 | 307 | 156 | 10 | 37 | 26 |
| NNC 63-0532 | + | 444 | 141 | 151 | 10 | 39 | 14 |
| NNC 711 | + | 351 | 180 | 101 | 10 | 25 | 16 |
| nocodazole | + | 302 | 111 | 146 | 10 | 47 | 14 |
| PHCCC | + | 295 | 161 | 111 | 10 | 41 | 14 |
| pilocarpine | + | 209 | 95 | 171 | 10 | 35 | 10 |
| PPT | + | 387 | 345 | 81 | 10 | 39 | 30 |
| PRE-084 | + | 318 | 114 | 56 | 10 | 25 | 12 |
| procaterol | + | 291 | 273 | 101 | 10 | 19 | 24 |
| PSB 11 | + | 296 | 242 | 191 | 10 | 37 | 20 |
| RHC 80267 | + | 395 | 114 | 111 | 10 | 15 | 12 |
| ritanserin | + | 478 | 193 | 76 | 10 | 39 | 16 |
| Ro 20-1724 | + | 279 | 223 | 141 | 10 | 15 | 20 |
| rolipram | + | 276 | 208 | 56 | 10 | 21 | 18 |
| RX 821002 | + | 235 | 165 | 76 | 10 | 29 | 16 |
| SB 200646 | + | 267 | 121 | 121 | 10 | 27 | 12 |
| SB 205384 | + | 331 | 261 | 151 | 10 | 37 | 22 |
| SB 218795 | + | 397 | 205 | 186 | 10 | 55 | 16 |
| SB 228357 | + | 432 | 215 | 61 | 10 | 39 | 18 |
| SB 297006 | + | 343 | 105 | 146 | 10 | 23 | 12 |
| SB 334867 | + | 320 | 146 | 91 | 10 | 21 | 12 |
| SB 366791 | + | 288 | 124 | 76 | 10 | 25 | 12 |
| SC 19220 | + | 332 | 232 | 101 | 10 | 15 | 18 |
| SCH 202676 | + | 268 | 180 | 56 | 10 | 35 | 16 |
| SDM25N | + | 415 | 98 | 171 | 10 | 33 | 12 |
| siguazodan | + | 285 | 269 | 196 | 10 | 31 | 22 |
| SKF 89976A | + | 336 | 129 | 156 | 10 | 33 | 12 |
| SKF 91488 | + | 176 | 100 | 21 | 10 | 15 | 10 |
| **Table S4 (continued)** |  |  |  |  |  |  |  |
| **compound** | **ESI** | **Q1**  **(Da)** | **Q3**  **(Da)** | **DP**  **(V)** | **EP**  **(V)** | **CE**  **(V)** | **CXP**  **(V)** |
| SMER 28 | + | 264 | 237 | 111 | 10 | 29 | 20 |
| SN-6 | + | 403 | 136 | 176 | 10 | 31 | 14 |
| SNC 80 | + | 450 | 296 | 71 | 10 | 19 | 26 |
| SR 59230A | + | 326 | 196 | 86 | 10 | 17 | 18 |
| SR 95531 | + | 288 | 159 | 96 | 10 | 51 | 14 |
| TC-O 9311 | + | 366 | 197 | 76 | 10 | 15 | 14 |
| thioperamide | + | 293 | 152 | 131 | 10 | 23 | 14 |
| tiagabine | + | 376 | 247 | 171 | 10 | 27 | 20 |
| TMS | + | 301 | 177 | 61 | 10 | 23 | 16 |
| tranilast | + | 328 | 191 | 66 | 10 | 13 | 18 |
| WIN 64338 | + | 356 | 106 | 71 | 10 | 39 | 12 |
| xamoterol | + | 340 | 253 | 101 | 10 | 21 | 22 |
| YM 976 | + | 314 | 286 | 86 | 10 | 35 | 30 |
| zaprinast | + | 272 | 77 | 71 | 10 | 75 | 10 |
| ZD 7288 | + | 258 | 157 | 76 | 10 | 41 | 18 |
| ZM 241385 | + | 338 | 176 | 116 | 10 | 39 | 18 |
| β-funaltrexamine | + | 455 | 308 | 166 | 10 | 39 | 26 |

**Table S5:** IC_50_ to p*K_i_* transformation and calculations of the equilibrium binding concentrations.

| **IC_50_** | ***K_d_* reporter ligand** | **[L]** | ***K_i_*** |
| --- | --- | --- | --- |
| 1000 nM | 23.6 nM | 10 nM | 702 nM |

$$\boldsymbol{K}_{\boldsymbol{i}}\boldsymbol{=}\frac{\boldsymbol{IC}_{\boldsymbol{50}}}{\boldsymbol{1}\boldsymbol{+}\frac{\boldsymbol{[}\boldsymbol{L}\boldsymbol{]}}{\boldsymbol{K}_{\boldsymbol{d}}}}$$

**Cheng-Prusoff-equation** with *K*_i_: affinity constant of inhibitor, IC_50_: half maximal inhibitory concentration, [L]: ligand concentration, *K_d_*: ligand dissociation constant

| **library constellation** | **L [nM]** | ***K_d_* ligand [nM]** | ***K_d_***  **(reporter ligand)**  **[nM]** | **I**  **(reporter ligand)**  **[nM]** | ***K_d_***  **(additional ligand)**  **[nM]** | **I (additional ligand)**  **[nM]** | **T**  **[nM]** | **L_bound_**  **[nM]** |
| --- | --- | --- | --- | --- | --- | --- | --- | --- |
| one ligand with *K_d_* of 702 nM | 1000 | 702 | 23.6 | 10 | - | - | 3 | 1.50 |
| two ligands with *K_d_* of 702 nM | 1000 | 702 | 23.6 | 10 | 702 | 1000 | 3 | 1.00 |
| two ligands with *K_d_* of 702 and 7 nM  (calculation for the low affinity ligand) | 1000 | 702 | 23.6 | 10 | 7 | 1000 | 3 | 0.03 |

$$\boldsymbol{L}_{\boldsymbol{bound}}\boldsymbol{=}\frac{\boldsymbol{L}}{\boldsymbol{K}_{\boldsymbol{d}}\boldsymbol{*}\left( \boldsymbol{1}\boldsymbol{+}\frac{\boldsymbol{I}_{\boldsymbol{(}\boldsymbol{reporter} \boldsymbol{ligand}\boldsymbol{)}}}{{\boldsymbol{K}_{\boldsymbol{d}}}_{\boldsymbol{(}\boldsymbol{reporter} \boldsymbol{ligand}\boldsymbol{)}}} \right)\boldsymbol{+}\boldsymbol{L}}\boldsymbol{*}\boldsymbol{T}$$

$$\boldsymbol{L}_{\boldsymbol{bound}}\boldsymbol{=}\frac{\boldsymbol{L}}{\boldsymbol{K}_{\boldsymbol{d}}\boldsymbol{*}\left( \boldsymbol{1}\boldsymbol{+}\frac{\boldsymbol{I}_{\boldsymbol{(}\boldsymbol{reporter} \boldsymbol{ligand}\boldsymbol{)}}}{{\boldsymbol{K}_{\boldsymbol{d}}}_{\boldsymbol{(}\boldsymbol{reporter} \boldsymbol{ligand}\boldsymbol{)}}}\boldsymbol{+}\frac{\boldsymbol{I}_{\boldsymbol{(}\boldsymbol{additional} \boldsymbol{ligand}\boldsymbol{)}}}{\boldsymbol{K}_{\boldsymbol{d}_{\boldsymbol{(}\boldsymbol{additional} \boldsymbol{ligand}\boldsymbol{)}}}} \right)\boldsymbol{+}\boldsymbol{L}}\boldsymbol{*}\boldsymbol{T}$$

L_bound_: concentration of bound ligand of interest, T: concentration of target, L: applied concentration of the ligand of interest, *K_d_*: dissociation constant of the ligand of interest, I_(reporter ligand)_: applied concentration of reporter ligand, *K*_d_: dissociation constant of reporter ligand, I_(additional ligand)_: applied concentration of an additional ligand, *K*_d_: dissociation constant of an additional ligand

**Table S6:** Results obtained for components of the deliberately compiled library from MRM chromatograms. Reported values represent means ± SD obtained from 1nM matrix standards (n = 3).

|  | **compound** | **t_R_** | **RRF** | **S/N** |  | **compound** | **t_R_** | **RRF** | **S/N** |
| --- | --- | --- | --- | --- | --- | --- | --- | --- | --- |
| A | 4-(4-chloro-phenyl)-piperidin-4-ol | n.d. | n.d. | n.d. | B | bifonazole | n.d. | n.d. | n.d. |
|  | chlorpromazine | 2.63 | 0.053 | 325 |  | ciprofloxacin | 0.82 | 0.266 | 96 |
|  | 8-OH DAPT | 0.90 | 1.187 | 5570 |  | DDPM3138 | 0.94 | 0.347 | 6520 |
|  | doxepin | 1.86 | 0.309 | 633 |  | glibenclamide | 1.50 | 0.005 | 93 |
|  | fenoterol | 0.68 | 0.288 | 1360 |  | DDPM2188 | 1.21 | 0.086 | 1180 |
|  | DDPM2565 | 1.01 | 0.095 | 1460 |  | hydromorphone | 0.69 | 0.094 | 1100 |
|  | ketoprofen | 0.74 | 0.020 | 117 |  | lisinopril | 0.44 | 0.009 | 121 |
|  | meclozine | 4.85 | 0.014 | 107 |  | molsidomine | 0.72 | 0.088 | 1370 |
|  | metoclopramide | 0.74 | 1.263 | 7680 |  | noscapine | 2.59 | 0.213 | 7480 |
|  | oxazepam | 1.19 | 0.159 | 818 |  | pilocarpine | 0.74 | 0.197 | 875 |
|  | piroxicam | 0.73 | 0.217 | 1930 |  | procainamide | 0.55 | 0.760 | 1560 |
|  | procaine | 0.72 | 0.407 | 3240 |  | prophyphenazone | 1.30 | 0.305 | 705 |
|  | roxithromycin | 2.17 | 0.001 | 58 |  | reserpine | 2.73 | 0.068 | 1060 |
|  | sulpiride | 0.68 | 0.240 | 1070 |  | sulfaguanidine | 0.50 | 0.010 | 107 |
|  | telmisartan | 0.92 | 0.608 | 1790 |  | sulfamethoxazole | 0.74 | 0.015 | 113 |
|  | DDPM2330 | 1.79 | 0.098 | 1040 |  | triphenylamine | - | - |  |
| C | (2-morpholin-4-ylmethyl-benzoimidazol-1-yl)-acetic acid | 0.98 | 0.144 | 981 | D | ambroxol | 1.52 | 0.082 | 919 |
|  | aciclovir | 2.48 | 0.015 | 26 |  | antazoline | 1.03 | 1.903 | 4060 |
|  | amitriptyline | 0.72 | 0.135 | 742 |  | biperiden | 2.41 | 1.417 | 4120 |
|  | atropine | 0.75 | 0.388 | 2373 |  | clotrimazole | n.d. | n.d. | n.d. |
|  | brucine | 1.01 | 0.050 | 1070 |  | diphenhydramine | 1.42 | 0.170 | 987 |
|  | cetirizine | 2.60 | 0.411 | 725 |  | diltiazem | 1.90 | 0.260 | 173 |
|  | clomipramine | 0.96 | 0.006 | 92 |  | DDPM2187 | 1.67 | 0.463 | 1620 |
|  | ethacridine | 0.92 | 0.157 | 88 |  | lidocaine | 1.71 | 1.893 | 3200 |
|  | indometacin | 0.95 | 0.012 | 280 |  | meloxicam | 0.78 | 0.078 | 1360 |
|  | mepivacaine | 0.85 | 0.852 | 5600 |  | ofloxacin | 0.79 | 0.589 | 450 |
|  | DDPM2077 | 0.47 | 0.162 | 1770 |  | physostigmine | 0.79 | 0.182 | 1540 |
|  | DDPM2029 | 0.87 | 0.220 | 2080 |  | propranolol | 1.03 | 0.275 | 863 |
|  | papaverine | 1.41 | 0.597 | 5070 |  | ranitidine | 0.68 | 0.133 | 1370 |
|  | promethazine | 2.56 | 0.193 | 1190 |  | sulfisomidine | 0.53 | 0.102 | 702 |
|  | salbutamol | 0.66 | 0.324 | 765 |  | tianeptine | 0.99 | 0.102 | 2350 |
|  | sertraline | 2.59 | 0.007 | 161 |  | DDPM2473 | 0.94 | 0.355 | 7040 |
| E | ASP+ | 1.15 | 0.158 | 360 | F | 2-(4-methyl-1,4-diazepan-1-yl)benzoic acid | 0.51 | 0.101 | 523 |
|  | atenolol | 0.51 | 0.093 | 266 |  | baclofen | 0.64 | 0.003 | 14 |
|  | benzylpenicillin | 0.44 | 0.003 | 60 |  | chloroquine | 3.10 | 0.353 | 261 |
|  | chlordiazepoxide | 1.28 | 0.030 | 121 |  | diazepam | 2.31 | 0.221 | 2650 |
|  | drofenine | 2.88 | 0.099 | 1410 |  | ditolylguanidine | 0.94 | 0.644 | 1500 |
|  | isoprenaline | 0.97 | 0.005 | 80 |  | haloperidol | 1.74 | 0.485 | 752 |
|  | DDPM1981 | 0.93 | 0.347 | 3280 |  | ipratropium | 0.72 | 0.079 | 1150 |
|  | meprobamate | 0.79 | 0.010 | 139 |  | metoprolol | 0.75 | 0.398 | 3860 |
|  | DDPM1349 | 0.97 | 0.273 | 3060 |  | moxifloxacin | 0.88 | 0.108 | 62 |
|  | morphine | 0.68 | 0.046 | 621 |  | DDPM2009 | 1.01 | 0.501 | 7230 |
|  | nalidixic acid | 0.99 | 0.909 | 371 |  | naphazoline | 0.77 | 0.696 | 1530 |
|  | phenylbutazone | 0.84 | 0.031 | 307 |  | phenobarbital | n.d. | n.d. | n.d. |
|  | terfenadine | 2.71 | 0.032 | 273 |  | pimozide | 4.95 | 0.024 | 76 |
|  | tetracaine | 1.64 | 0.745 | 2690 |  | scopolamine | 0.68 | 0.513 | 6470 |
|  | tolbutamide | 0.82 | 0.007 | 109 |  | tetracycline | 0.75 | 0.046 | 52 |
|  | verapamil | 2.24 | 0.043 | 79 |  | tramadol | 0.80 | 0.671 | 1510 |
|  |  |  |  |  |  |  |  |  |  |
|  | **Table S6 (continued)** | | | |  |  |  |  |  |
|  | **compound** | **t_R_** | **RRF** | **S/N** |  | **compound** | **t_R_** | **RRF** | **S/N** |
| G | buspirione | 1.93 | 0.175 | 797 | H | 3-(2-methyl-1H-imidazol-1-yl)benzoic acid | 0.59 | 0.031 | 47 |
|  | quinine | 1.04 | 0.159 | 579 |  | acetazolamide | 0.67 | 0.084 | 43 |
|  | clonidine | 0.77 | 0.019 | 313 |  | captopril | 0.45 | 0.001 | 5 |
|  | cimetidine | 0.68 | 0.109 | 528 |  | chloramphenicol | 0.85 | 0.261 | 1740 |
|  | DDPM3139 | 2.90 | 0.004 | 58 |  | chlortalidone | 0.76 | 0.036 | 463 |
|  | imipramine | 2.34 | 0.461 | 2050 |  | diclofenac | 0.90 | 0.013 | 49 |
|  | metipranolol | 0.91 | 0.087 | 558 |  | etacrynic acid | 0.82 | 0.016 | 65 |
|  | PH014034 | 2.98 | 0.074 | 632 |  | furosemide | 0.73 | 0.008 | 34 |
|  | piretanide | 0.77 | 0.012 | 494 |  | hydrochlorothiazide | 0.74 | 0.156 | 426 |
|  | ramipril | 0.79 | 0.810 | 11000 |  | mefenamic acid | 1.06 | 0.074 | 47 |
|  | riboflavin | n.d. | n.d. | n.d. |  | methyl orange | 0.83 | 0.376 | 3740 |
|  | strychnine nitrate | 0.82 | 0.206 | 1980 |  | naproxen | 0.72 | 0.033 | 5 |
|  | tiabendazole | 0.87 | 0.674 | 5300 |  | lauryl maltoside | 2.36 | 0.001 | 23 |
|  | trifluoperazine | 3.11 | 2.281 | 74 |  | niclosamide | 2.85 | 0.833 | 590 |
|  | trimethoprim | 0.73 | 0.303 | 2620 |  | nitrazepam | 1.26 | 0.711 | 2060 |
|  | xylometazoline | 1.48 | 0.326 | 906 |  | phenytoin | 1.10 | 0.010 | 36 |

n.d.: not detected

**Table S7:** Hit identification for the deliberately compiled library. Total and nonspecific binding of each sublibrary component at a concentration of 1µM. Nonspecific binding determined in presence of 100 mM GABA. Reported values represent means ± SD (n=3). Values marked red represent identified hits based on significantly higher total than nonspecific binding (one-tailed *t*-test, CL = 97.5%).

|  | **compound** | **total binding**  **[normalized area]** | **nonspecific binding [normalized area]** |  | **compound** | **total binding [normalized area]** | **nonspecific binding**  **[normalized area]** |
| --- | --- | --- | --- | --- | --- | --- | --- |
| A | 4-(4-chloro-phenyl)-piperidin-4-ol | n.d | n.d | B | bifonazole | n.d | n.d |
|  | chlorpromazine | 6.590±0.156 | 6.370±0.198 |  | ciprofloxacin | 0.046±0.003 | 0.048±0.002 |
|  | 8-OH DAPT | 0.177±0.006 | 0.193±0.004 |  | DDPM3138 | 0.533±0.038 | 0.226±0.023 |
|  | doxepin | 0.705±0.025 | 0.666±0.008 |  | glibenclamide | 0.018±0.003 | 0.020±0.004 |
|  | fenoterol | 0.041±0.008 | 0.041±0.002 |  | DDPM2188 | 0.133±0.008 | 0.118±0.004 |
|  | DDPM2565 | 0.571±0.040 | 0.119±0.006 |  | hydromorphone | 0.031±0.002 | 0.029±0.001 |
|  | ketoprofen | 0.004±0.000 | 0.005±0.000 |  | lisinopril | 0.001±0.000 | 0.001±0.000 |
|  | meclozine | 4.937±0.861 | 6.095±0.205 |  | molsidomine | 0.011±0.001 | 0.012±0.000 |
|  | metoclopramide | 0.370±0.013 | 0.390±0.028 |  | noscapine | 0.105±0.007 | 0.114±0.010 |
|  | oxazepam | 0.132±0.001 | 0.130±0.026 |  | pilocarpine | 0.114±0.008 | 0.092±0.004 |
|  | piroxicam | 0.054±0.000 | 0.060±0.005 |  | procainamide | 0.139±0.014 | 0.129±0.001 |
|  | procaine | 0.073±0.003 | 0.074±0.006 |  | prophyphenazone | 0.045±0.006 | 0.049±0.002 |
|  | roxithromycin | 0.020±0.003 | 0.021±0.001 |  | reserpine | 16.13±0.760 | 15.73±0.682 |
|  | sulpiride | 0.054±0.002 | 0.056±0.004 |  | sulfaguanidine | n.q. | n.q. |
|  | telmisartan | 3.853±0.130 | 3.405±0.106 |  | sulfamethoxazole | n.q. | n.q. |
|  | DDPM2330 | 0.771±0.003 | 0.738±0.002 |  | triphenylamine | n.d | n.d |
| C | (2-morpholin-4-ylmethyl-benzoimidazol-1-yl)-acetic acid | 0.042±0.002 | 0.039±0.004 | D | ambroxol | 0.076±0.002 | 0.072±0.004 |
|  | aciclovir | 3.470±0.339 | 3.495±0.219 |  | antazoline | 1.240±0.014 | 1.197±0.076 |
|  | amitriptyline | 1.077±0.090 | 1.075±0.064 |  | biperiden | 2.515±0.035 | 2.620±0.260 |
|  | atropine | 0.122±0.011 | 0.118±0.003 |  | clotrimazole | n.d | n.d |
|  | brucine | 0.037±0.003 | 0.038±0.001 |  | diphenhydramine | 0.114±0.004 | 0.115±0.012 |
|  | cetirizine | 0.491±0.049 | 0.562±0.092 |  | diltiazem | 2.330±0.028 | 2.390±0.352 |
|  | clomipramine | 0.338±0.021 | 0.333±0.021 |  | DDPM2187 | 1.150±0.035 | 1.050±0.046 |
|  | ethacridine | 32.07±1.850 | 31.60±0.141 |  | lidocaine | 0.436±0.049 | 0.405±0.013 |
|  | indometacin | 0.043±0.001 | 0.048±0.005 |  | meloxicam | 0.022±0.002 | 0.025±0.003 |
|  | mepivacaine | 0.185±0.023 | 0.184±0.003 |  | ofloxacin | 0.146±0.017 | 0.133±0.003 |
|  | DDPM2077 | 0.147±0.014 | 0.111±0.004 |  | physostigmine | 0.042±0.005 | 0.037±0.002 |
|  | DDPM2029 | 0.508±0.044 | 0.182±0.025 |  | propranolol | 0.173±0.014 | 0.156±0.009 |
|  | papaverine | 0.719±0.059 | 0.814±0.099 |  | ranitidine | 0.028±0.005 | 0.025±0.002 |
|  | promethazine | 1.173±0.110 | 1.195±0.049 |  | sulfisomidine | 0.012±0.000 | 0.010±0.001 |
|  | salbutamol | 0.082±0.006 | 0.072±0.012 |  | tianeptine | 0.064±0.012 | 0.053±0.002 |
|  | sertraline | 1.747±0.180 | 1.700±0.028 |  | DDPM2473 | 0.868±0.033 | 0.300±0.026 |
|  |  |  |  |  |  |  |  |
|  |  |  |  |  |  |  |  |
|  |  |  |  |  |  |  |  |
|  |  |  |  |  |  |  |  |
|  |  |  |  |  |  |  |  |
|  |  |  |  |  |  |  |  |
|  |  |  |  |  |  |  |  |
|  |  |  |  |  |  |  |  |
|  |  |  |  |  |  |  |  |
|  |  |  |  |  |  |  |  |
|  |  |  |  |  |  |  |  |
|  |  |  |  |  |  |  |  |
|  |  |  |  |  |  |  |  |
|  |  |  |  |  |  |  |  |
|  |  |  |  |  |  |  |  |
|  |  |  |  |  |  |  |  |
|  |  |  |  |  |  |  |  |
|  |  |  |  |  |  |  |  |
|  | **Table S7 (continued)** | | |  |  |  |  |
|  | **compound** | **total binding**  **[normalized area]** | **nonspecific binding [normalized area]** |  | **compound** | **total binding [normalized area]** | **nonspecific binding**  **[normalized area]** |
| E | ASP+ | 0.122±0.014 | 0.141±0.017 | F | 2-(4-methyl-1,4-diazepan-1-yl)benzoic acid | 0.012±0.002 | 0.011±0.001 |
|  | atenolol | 0.012±0.002 | 0.011±0.001 |  | baclofen | n.q. | n.q. |
|  | benzylpenicillin | n.q. | n.q. |  | chloroquine | 1.063±0.353 | 0.692±0.060 |
|  | chlordiazepoxide | 0.016±0.006 | 0.014±0.004 |  | diazepam | 0.134±0.042 | 0.086±0.009 |
|  | drofenine | 1.843±0.229 | 2.150±0.100 |  | ditolylguanidine | 0.140±0.038 | 0.105±0.006 |
|  | isoprenaline | 0.006±0.001 | 0.006±0.001 |  | haloperidol | 4.270±1.524 | 2.803±0.412 |
|  | DDPM1981 | 0.666±0.064 | 0.311±0.024 |  | ipratropium bromide | 0.018±0.005 | 0.012±0.001 |
|  | meprobamate | 0.002±0.001 | 0.002±0.000 |  | metoprolol | 0.068±0.009 | 0.057±0.003 |
|  | DDPM1349 | 0.290±0.016 | 0.265±0.004 |  | moxifloxacin | 0.034±0.011 | 0.028±0.006 |
|  | morphine | 0.008±0.001 | 0.009±0.000 |  | DDPM2009 | 1.053±0.277 | 0.364±0.048 |
|  | nalidixic acid | 0.605±0.045 | 0.525±0.070 |  | naphazoline | 0.173±0.033 | 0.127±0.008 |
|  | phenylbutazone | 0.012±0.001 | 0.012±0.000 |  | phenobarbital | n.d | n.d |
|  | terfenadine | 10.41±0.681 | 9.773±0.398 |  | pimozide | 14.10±2.081 | 12.33±1.604 |
|  | tetracaine | 0.702±0.079 | 0.812±0.050 |  | scopolamine | 0.075±0.007 | 0.065±0.005 |
|  | tolbutamide | n.q. | n.q. |  | tetracycline | n.q. | n.q. |
|  | verapamil | 1.057±0.155 | 1.137±0.061 |  | tramadol | 0.131±0.028 | 0.112±0.002 |
| G | buspirione | 0.014±0.001 | 0.013±0.001 | H | 3-(2-methyl-1H-imidazol-1-yl)benzoic acid | 0.001±0.000 | 0.002±0.000 |
|  | quinine | 0.120±0.016 | 0.109±0.022 |  | acetazolamide | 0.002±0.001 | 0.003±0.001 |
|  | clonidine | 0.002±0.000 | 0.002±0.000 |  | captopril | n.q. | n.q. |
|  | cimetidine | 0.015±0.000 | 0.014±0.001 |  | chloramphenicol | 0.022±0.002 | 0.027±0.005 |
|  | DDPM3139 | 0.282±0.022 | 0.265±0.031 |  | chlortalidone | 0.005±0.001 | 0.005±0.001 |
|  | imipramine | 1.153±0.127 | 1.117±0.055 |  | diclofenac | 0.009±0.000 | 0.010±0.002 |
|  | metipranolol | 0.046±0.009 | 0.039±0.010 |  | etacrynic acid | 0.018±0.001 | 0.015±0.005 |
|  | PH014034 | 13.03±1.762 | 14.30±0.964 |  | furosemide | 0.002±0.000 | 0.002±0.000 |
|  | piretanide | 0.003±0.001 | 0.003±0.001 |  | hydrochlorothiazide | 0.007±0.001 | 0.009±0.001 |
|  | ramipril | 1.064±0.284 | 0.659±0.359 |  | mefenamic acid | 0.053±0.003 | 0.053±0.004 |
|  | riboflavin | n.d | n.d |  | methyl orange | 0.453±0.052 | 0.427±0.057 |
|  | strychnine nitrate | 0.048±0.004 | 0.046±0.002 |  | naproxen | n.q. | n.q. |
|  | tiabendazole | 0.169±0.007 | 0.153±0.011 |  | lauryl maltoside | 0.082±0.006 | 0.071±0.006 |
|  | trifluoperazine | 7.503±0.662 | 5.953±0.510 |  | niclosamide | 34.84±3.522 | 29.38±4.819 |
|  | trimethoprim | 0.051±0.003 | 0.046±0.002 |  | nitrazepam | 0.174±0.041 | 0.159±0.027 |
|  | xylometazoline | 0.417±0.046 | 0.424±0.069 |  | phenytoin | 0.002±0.000 | 0.002±0.000 |

n.d.: not detected; n.q.: binding below the lower limit of quantification

**Table S8:** Remaining NO711 binding determined by means of gradient LC-ESI-MS/MS. NO711 binding in % in presence of the sublibrary A-H (concentration 1µM each compound) using a Purospher Star RP18e column (55 mm × 2 mm, 3 μm) in combination with 10 mM ammonium formate buffer (pH 7.0) and acetonitrile (gradient conditions see “materials and methods” at a column temperature of 25°C, flow rate of 450 μL/min and an injection volume of 40 µL.

| **sublibrary** | **A** | **B** | **C** | **D** | **E** | **F** | **G** | **H** |
| --- | --- | --- | --- | --- | --- | --- | --- | --- |
| **NO711 binding %** | 1.5 | 2.6 | 26.7 | 2.0 | 4.5 | 47.7 | 87.7 | 58.1 |

**Table S9:** Results obtained for components of the Tocris Screen Plus library from MRM chromatograms. Reported values represent means ± SD obtained from 1nM matrix standards (n = 3).

| **Nr** | **compound** | **t_R_** | **RRF** | **S/N** | **Nr** | **compound** | **t_R_** | **rRF** | **S/N** |
| --- | --- | --- | --- | --- | --- | --- | --- | --- | --- |
| 5 | (-)-U-50488 | 1.61 | 0.374 | 679 | 9 | 8-M-PDOT | 1.13 | 1.180 | 2280 |
|  | 3'-fluorobenzylspiperone | 2.65 | 0.008 | 123 |  | AF-DX 116 | 0.80 | 1.450 | 2120 |
|  | BNTX | 1.00 | 0.283 | 1427 |  | BD 1047 | 1.69 | 0.199 | 158 |
|  | castanospermine | 0.47 | 0.106 | 92 |  | CGP 7930 | 0.85 | 0.020 | 21 |
|  | GBR 12909 | 2.85 | 0.008 | 166 |  | CI 966 | 1.13 | 0.985 | 1960 |
|  | L-732,138 | 2.79 | 0.001 | 30 |  | DH 97 | 2.44 | 0.060 | 174 |
|  | MDL 11,939 | 0.92 | 1.433 | 1077 |  | DuP 697 | n.d. | n.d. | n.d. |
|  | MDL 72222 | 1.76 | 0.177 | 238 |  | GR 159897 | 0.93 | 0.140 | 501 |
|  | methiothepin | 2.62 | 0.065 | 361 |  | MRS 1220 | 2.90 | 0.082 | 520 |
|  | pilocarpine | 0.74 | 0.147 | 789 |  | PPT | 1.13 | 0.032 | 104 |
|  | PRE-084 | 2.80 | 0.174 | 2810 |  | procaterol | 0.59 | 0.258 | 10 |
|  | Ro 20-1724 | 1.16 | 0.096 | 137 |  | SB 205384 | 1.05 | 0.951 | 2490 |
|  | rolipram | 0.98 | 0.242 | 2700 |  | SB 218795 | 2.84 | 0.020 | 164 |
|  | SKF 91488 | 0.75 | 0.021 | 44 |  | SB 228357 | 2.84 | 2.000 | 9850 |
|  | SNC 80 | 2.08 | 0.058 | 1407 |  | xamoterol | 0.59 | 0.619 | 745 |
|  | thioperamide | 0.88 | 0.223 | 545 |  | ZM 241385 | 0.84 | 0.043 | 176 |
| 6 | brefeldin A | n.d. | n.d. | n.d. | 14 | apoptosis activator 2 | 2.63 | 0.011 | 27 |
|  | BW 723C86 | 0.97 | 0.077 | 107 |  | cisapride | 1.20 | 0.809 | 351 |
|  | cilostamide | 1.20 | 0.047 | 970 |  | CMPD-1 | 2.12 | 0.366 | 1270 |
|  | clemastine | 2.58 | 0.039 | 413 |  | DAU 5884 | 0.79 | 0.972 | 933 |
|  | CP 94253 | 0.84 | 0.447 | 879 |  | IEM 1460 | n.d. | n.d. | n.d. |
|  | dibutyryl-cAMP | 0.63 | 0.003 | 227 |  | L-655,240 | 2.65 | 0.013 | 30 |
|  | formoterol | 0.68 | 0.471 | 1317 |  | mirtazapine | 1.28 | 2.400 | 1390 |
|  | FPL 64176 | 2.68 | 0.081 | 2033 |  | NNC 63-0532 | 3.20 | 0.826 | 6050 |
|  | KU14R | 2.58 | 0.060 | 333 |  | NNC 711 | 0.83 | 1.120 | 1870 |
|  | L-701,324 | 2.53 | 0.034 | 217 |  | PSB 11 | 0.86 | 1.190 | 3310 |
|  | LY 225910 | 3.18 | 0.001 | 114 |  | RHC 80267 | 1.83 | 0.005 | 64 |
|  | nocodazole | 1.03 | 0.223 | 2260 |  | ritanserin | 2.4 | 1.100 | 2890 |
|  | RX 821002 | 0.669 | 0.061 | 293 |  | SB 334867 | 0.99 | 1.550 | 2660 |
|  | SCH 202676 | 0.948 | 0.007 | 32 |  | SB 366791 | 2.59 | 0.039 | 76 |
|  | WIN 64338 | 2.87 | 0.026 | 20 |  | SN-6 | 3.00 | 0.007 | 28 |
|  | ZD 7288 | 1.15 | 0.035 | 95 |  | YM 976 | 2.57 | 0.075 | 161 |
| 7 | (-)-quinpirole | 0.74 | 0.465 | 520 | 38 | 4E1RCat | 1.18 | 0.010 | 13 |
|  | (+)-MK 801 | 0.95 | 1.690 | 2280 |  | A 582941 | 0.88 | 1.140 | 707 |
|  | (R)-(-)-rolipram | 0.99 | 0.191 | 194 |  | ADX 10059 | 1.69 | 0.767 | 585 |
|  | CGP 55845 | 0.87 | 0.035 | 725 |  | AT 1015 | 1.14 | 0.053 | 127 |
|  | CPCCOEt | 1.19 | 0.011 | 176 |  | BIMU 8 | 0.99 | 4.110 | 1220 |
|  | daidzein | 0.84 | 0.017 | 7 |  | calhex 231 | 2.83 | 0.621 | 2520 |
|  | flecainide | 0.96 | 0.479 | 745 |  | DBeQ | 2.84 | 0.81 | 198 |
|  | GYKI 52466 | 0.89 | 1.170 | 2490 |  | EMD 281014 | 1.48 | 0.003 | 17 |
|  | KB-R7943 | 1.10 | 0.922 | 174 |  | FH 535 | 1.26 | 0.037 | 202 |
|  | L-655,708 | 0.86 | 0.096 | 104 |  | flurizan | n.d. | n.d. | n.d. |
|  | PHCCC | 0.88 | 0.143 | 10 |  | ICA 069673 | 1.41 | 0.034 | 105 |
|  | SC 19220 | 1.03 | 0.460 | 1030 |  | ML 218 | 1.70 | 0.597 | 546 |
|  | SDM25N | 0.96 | 0.479 | 745 |  | SB 297006 | 1.83 | 0.044 | 91 |
|  | siguazodan | 0.79 | 0.022 | 501 |  | SMER 28 | 1.33 | 0.861 | 1820 |
|  | SKF 89976A | 0.85 | 1.500 | 2120 |  | TC-O 9311 | 1.12 | 0.005 | 18 |
|  | β-funaltrexamine | 0.80 | 0.432 | 158 |  | tiagabine | 0.88 | 0.898 | 2020 |
| 8 | 4-P-PDOT | 2.53 | 0.102 | 759 |  |  |  |  |  |
|  | CGP 54626 | 6.07 | 0.067 | 30 |  |  |  |  |  |
|  | DCEBIO | 1.75 | 0.010 | 25 |  |  |  |  |  |
|  | FR 139317 | 1.15 | 0.131 | 3200 |  |  |  |  |  |
|  | methyllycaconitine | 0.873 | 0.146 | 2513 |  |  |  |  |  |
|  | MG 624 | 2.41 | 0.062 | 406 |  |  |  |  |  |
|  | MPEP | 2.63 | 0.085 | 788 |  |  |  |  |  |
|  | naloxone benzoylhydrazone | 0.735 | 0.033 | 636 |  |  |  |  |  |
|  | olvanil | n.d. | n.d. | n.d. |  |  |  |  |  |
|  | SB 200646 | 0.946 | 0.273 | 922 |  |  |  |  |  |
|  | SDZ 220-581 | n.d. | n.d. | n.d. |  |  |  |  |  |
|  | SR 59230A | 2.34 | 0.103 | 494 |  |  |  |  |  |
|  | SR 95531 | 0.665 | 0.088 | 381 |  |  |  |  |  |
|  | TMS | 0.58 | 0.010 | 50 |  |  |  |  |  |
|  | tranilast | 0.653 | 0.002 | 72 |  |  |  |  |  |

n.d.: not detected

**Table S10:** Hit identification of Tocris Screen Plus library (16 membered sublibraries). Total and nonspecific binding of each sublibrary component at a concentration of 1µM. Nonspecific binding determined in presence of 100 mM GABA. Reported values represent means ± SD (n=3). Values marked red represent identified hits based on significantly higher total than nonspecific binding (one-tailed *t*-test, CL = 97.5%).

| **Nr** | **compound** | **total binding**  **[normalized area]** | **nonspecific binding [normalized area]** | **Nr** | **compound** | **total binding [normalized area]** | **nonspecific binding**  **[normalized area]** |
| --- | --- | --- | --- | --- | --- | --- | --- |
| 7 | (-)-quinpirole | 0.059±0.012 | 0.057±0.004 | 9 | 8-M-PDOT | 0.460±0.117 | 0.502±0.149 |
|  | (+)-MK 801 | 0.291±0.091 | 0.294±0.051 |  | AF-DX 116 | 0.835±0.043 | 0.833±0.099 |
|  | (R)-(-)-rolipram | 0.029±0.009 | 0.028±0.008 |  | BD 1047 | 1.470±0.305 | 1.783±0.305 |
|  | CGP 55845 | 0.018±0.005 | 0.018±0.003 |  | CGP 7930 | 0.024±0.002 | 0.027±0.008 |
|  | CPCCOEt | 0.005±0.001 | 0.005±0.001 |  | CI 966 | 5.944±0.339 | 4.553±0.333 |
|  | daidzein | 0.003±0.001 | 0.003±0.001 |  | DH 97 | 29.41±9.231 | 32.65±8.500 |
|  | flecainide | 0.646±0.120 | 0.560±0.097 |  | DuP 697 | n.d. | n.d. |
|  | GYKI 52466 | 0.317±0.066 | 0.320±0.082 |  | GR 159897 | 1.017±0.206 | 1.143±0.105 |
|  | KB-R7943 | 13.60±3.628 | 14.68±3.330 |  | MRS 1220 | 45.70±2.152 | 43.56±3.327 |
|  | L-655,708 | 0.030±0.007 | 0.032±0.008 |  | PPT | 34.73±3.92 | 36.90±3.799 |
|  | PHCCC | 0.038±0.007 | 0.038±0.010 |  | procaterol | 0.051±0.004 | 0.055±0.012 |
|  | SC 19220 | 0.014±0.003 | 0.013±0.002 |  | SB 205384 | 62.35±5.322 | 60.70±7.438 |
|  | SDM25N | 3.592±0.437 | 3.808±0.601 |  | SB 218795 | 34.15±6.924 | 35.91±5.864 |
|  | siguazodan | n.q. | n.q. |  | SB 228357 | 50.02±2.252 | 50.53±6.380 |
|  | SKF 89976A | 2.093±0.160 | 0.532±0.034 |  | xamoterol | 0.060±0.007 | 0.061±0.005 |
|  | β-funaltrexamine | 0.068±0.017 | 0.066±0.004 |  | ZM 241385 | 0.429±0.024 | 0.417±0.115 |
| 14 | apoptosis activator 2 | 0.028±0.006 | 0.047±0.022 | 38 | 4E1RCat | 0.715±0.144 | 0.831±0.122 |
|  | cisapride | 5.390±1.417 | 4.555±1.728 |  | A 582941 | 1.135±0.234 | 1.277±0.114 |
|  | CMPD-1 | 0.264±0.032 | 0.275±0.048 |  | ADX 10059 | 1.533±0.254 | 1.563±0.225 |
|  | DAU 5884 | 0.213±0.029 | 0.233±0.014 |  | AT 1015 | 5.310±0.792 | 5.337±0.729 |
|  | IEM 1460 | n.d. | n.d. |  | BIMU 8 | 6.347±1.282 | 7.270±0.551 |
|  | L-655,240 | 0.484±0.114 | 0.675±0.099 |  | calhex 231 | 29.23±5.350 | 26.86±5.863 |
|  | mirtazapine | 0.939±0.096 | 1.080±0.268 |  | DBeQ | 52.87±11.83 | 55.06±7.706 |
|  | NNC 63-0532 | 24.10±2.265 | 24.13±3.963 |  | EMD 281014 | 0.063±0.010 | 0.063±0.006 |
|  | NNC 711 (NO711) | 1.937±0.040 | 0.410±0.022 |  | FH 535 | 5.550±1.364 | 7.426±1.608 |
|  | PSB 11 | 0.413±0.144 | 0.502±0.139 |  | flurizan | n.d. | n.d. |
|  | RHC 80267 | 0.027±0.006 | 0.030±0.010 |  | ICA 069673 | 0.019±0.004 | 0.021±0.005 |
|  | ritanserin | 79.17±6.801 | 74.33±2.801 |  | ML 218 | 4.833±0.940 | 5.307±0.755 |
|  | SB 334867 | 5.150±0.976 | 6.217±1.913 |  | SB 297006 | 0.009±0.001 | 0.009±0.002 |
|  | SB 366791 | 21.92±5.719 | 25.53±5.243 |  | SMER 28 | 0.300±0.049 | 0.339±0.046 |
|  | SN-6 | 2.983±1.860 | 2.573±1.155 |  | TC-O 9311 | 0.181±0.025 | 0.196±0.044 |
|  | YM 976 | 0.345±0.040 | 0.385±0.084 |  | tiagabine | 2.140±0.104 | 0.768±0.071 |

n.d.: not detected; n.q.: binding below the lower limit of quantification

**Table S11:** Hit identification of Tocris Screen Plus library (64 membered sublibrary).Total and nonspecific binding of each sublibrary component at a concentration of 1µM. Nonspecific binding determined in presence of 100 mM GABA. Reported values represent means ± SD (n=3). Values marked red represent identified hits based on significantly higher total than nonspecific binding (one-tailed *t*-test, CL = 97.5%).

| **compound** | **total binding**  **[normalized area]** | **nonspecific binding [normalized area]** | **compound** | **total binding**  **[normalized area]** | **nonspecific binding [normalized area]** |
| --- | --- | --- | --- | --- | --- |
| (-)-U-50488 | 2.717±0.040 | 2.610±0.358 | (-)-quinpirole | 0.044±0.008 | 0.039±0.006 |
| 3'-fluorobenzylspiperone | 22.500±4.258 | 19.30±0.200 | (+)-MK 801 | 0.582±0.039 | 0.554±0.057 |
| BNTX | 4.477±0.136 | 4.073±0.505 | (R)-(-)-rolipram | 0.087±0.005 | 0.083±0.007 |
| castanospermine | 0.298±0.012 | 0.271±0.036 | CGP 55845 | 0.119±0.004 | 0.103±0.019 |
| GBR 12909 | 7.050±0.151 | 6.977±0.551 | CPCCOEt | 0.002±0.000 | 0.002±0.000 |
| L-732,138 | 0.432±0.040 | 0.496±0.020 | daidzein | 0.980±0.005 | 1.089±0.185 |
| MDL 11,939 | 1.660±0.070 | 1.647±0.189 | flecainide | 8.617±0.369 | 8.970±0.125 |
| MDL 72222 | 1.393±0.012 | 1.360±0.141 | GYKI 52466 | 0.535±0.034 | 0.531±0.016 |
| methiothepin | 33.533±0.513 | 34.53±2.268 | KB-R7943 | 27.067±0.757 | 27.367±0.416 |
| pilocarpine | 0.045±0.000 | 0.041±0.003 | L-655,708 | 0.098±0.007 | 0.094±0.010 |
| PRE-084 | 1.350±0.101 | 1.147±0.186 | PHCCC | 0.062±0.007 | 0.055±0.008 |
| Ro 20-1724 | 0.042±0.002 | 0.040±0.005 | SC 19220 | 0.020±0.003 | 0.020±0.002 |
| rolipram | 0.089±0.010 | 0.089±0.013 | SDM25N | 0.789±0.019 | 0.816±0.085 |
| SKF 91488 | n.q. | n.q | siguazodan | 0.005±0.001 | 0.004±0.000 |
| SNC 80 | 3.727±0.341 | 3.350±0.357 | SKF 89976A | 2.020±0.092 | 1.150±0.066 |
| Thioperamide | 0.874±0.021 | 0.870±0.089 | β-funaltrexamine | 0.155±0.018 | 0.144±0.023 |
| brefeldin A | n.d. | n.d. | 4-P-PDOT | 0.100±0.007 | 0.097±0.014 |
| BW 723C86 | 0.773±0.022 | 0.730±0.063 | CGP 54626 | 1.140±0.089 | 1.080±0.101 |
| cilostamide | 1.170±0.161 | 1.092±0.178 | DCEBIO | 0.008±0.001 | 0.007±0.001 |
| clemastine | 3.973±0.404 | 4.167±0.285 | FR 139317 | 1.427±0.076 | 1.280±0.145 |
| CP 94253 | 2.853±0.162 | 2.837±0.233 | methyllycaconitine | 0.403±0.048 | 0.342±0.044 |
| dibutyryl-cAMP | n.q. | n.q. | MG 624 | 6.043±0.433 | 6.190±0.185 |
| formoterol | 0.413±0.028 | 0.414±0.044 | MPEP | 0.301±0.009 | 0.279±0.029 |
| FPL 64176 | 3.770±0.312 | 3.733±0.125 | naloxone benzoylhydrazone | 0.192±0.011 | 0.166±0.023 |
| KU14R | 6.067±0.193 | 6.053±0.296 | olvanil | n.d. | n.d. |
| L-701,324 | 5.940±0.297 | 5.923±0.311 | SB 200646 | 0.432±0.033 | 0.425±0.025 |
| LY 225910 | 5.840±0.217 | 4.653±0.784 | SDZ 220-581 | n.d. | n.d. |
| nocodazole | 8.287±0.845 | 8.360±0.976 | SR 59230A | 15.767±0.603 | 15.867±0.306 |
| RX 821002 | 0.035±0.004 | 0.036±0.006 | SR 95531 | 0.026±0.002 | 0.025±0.003 |
| SCH 202676 | n.q. | n.q. | TMS | 0.019±0.000 | 0.020±0.003 |
| WIN 64338 | 41.67±2.146 | 38.23±3.353 | tranilast | n.q. | n.q |
| ZD 7288 | 0.052±0.003 | 0.047±0.007 | zaprinast | 0.009±0.002 | 0.011±0.002 |

n.d.: not detected; n.q.: binding below the lower limit of quantification
